# Lifestyle factors and their relative contributions to longitudinal progression of cardio-renal-metabolic multimorbidity: a prospective cohort study

**DOI:** 10.1186/s12933-024-02347-3

**Published:** 2024-07-18

**Authors:** Ning Zhang, Xiang Liu, Lele Wang, Yuan Zhang, Yi Xiang, Jiajie Cai, Hao Xu, Xiong Xiao, Xing Zhao

**Affiliations:** 1 West China School of Public Health and West China Fourth Hospital, Sichuan University, Chengdu, 610041 China; 2 State Key Laboratory of Oral Diseases, National Clinical Research Center for Oral Diseases, Research Unit of Oral Carcinogenesis and Management, West China Hospital of Stomatology, Chinese Academy of Medical Sciences , Sichuan University, Chengdu, 610041 China

**Keywords:** Lifestyle, Cardiovascular diseases, Diabetes, Chronic kidney disease, Multimorbidity

## Abstract

**Background:**

The role of lifestyle factors and their relative contributions to the development and mortality of cardio-renal-metabolic multimorbidity (CRMM) remains unclear.

**Methods:**

A study was conducted with 357,554 UK Biobank participants. CRMM was defined as the coexistence of two or three cardio-renal-metabolic diseases (CRMDs), including cardiovascular disease (CVD), type 2 diabetes (T2D) and chronic kidney disease (CKD). The prospective study examined the associations of individual and combined lifestyle scores (diet, alcohol consumption, smoking, physical activity, sedentary behavior, sleep duration and social connection) with longitudinal progression from healthy to first cardio-renal-metabolic disease (FCRMD), then to CRMM, and ultimately to death, using a multistate model. Subsequently, quantile G-computation was employed to assess the relative contribution of each lifestyle factor.

**Results:**

During a median follow-up of 13.62 years, lifestyle played crucial role in all transitions from healthy to FCRMD, then to CRMM, and ultimately to death. The hazard ratios (95% CIs) per score increase were 0.91 (0.90, 0.91) and 0.90 (0.89, 0.91) for healthy to FCRMD, and for FCRMD to CRMM, and 0.84 (0.83, 0.86), 0.87 (0.86, 0.89), and 0.90 (0.88, 0.93) for mortality risk from healthy, FCRMD, and CRMM, respectively. Among the seven factors, smoking status contributed to high proportions for the whole disease progression, accounting for 19.88–38.10%. High-risk diet contributed the largest proportion to the risk of transition from FCRMD to CRMM, with 22.53%. Less-frequent social connection contributed the largest proportion to the risk of transition from FCRMD to death, with 28.81%. When we further consider the disease-specific transitions, we find that lifestyle scores had slightly stronger associations with development to T2D than to CVD or CKD.

**Conclusions:**

Our study indicates that a healthy lifestyle may have a protective effect throughout the longitudinal progression of CRMM, informing more effective management and treatment. Smoking status, diet, and social connection played pivotal roles in specific disease transitions.

**Supplementary Information:**

The online version contains supplementary material available at 10.1186/s12933-024-02347-3.

## Introduction

Cardio-renal-metabolic conditions are leading causes of death and disability, with 1 in 3 deaths attributed to cardiovascular disease (CVD), type 2 diabetes (T2D) or chronic kidney disease (CKD) [1–7]. Growing evidence shows that there are close physiological links between CVD, T2D and CKD, so that they frequently coexist [3, 6]. Cardio-renal-metabolic multimorbidity (CRMM) was defined as the coexistence of two or three cardio-renal-metabolic diseases (CRMDs), including CVD, T2D and CKD [1, 3, 8–10]. With prolonged life expectancy and improved chronic disease management, individuals now are more likely to further develop CRMM after first cardio-renal-metabolic disease (FCRMD), which has attracted extensive attention from international organizations, such as the American Heart Association (AHA) [1–3, 6, 11–13].

Previous studies have predominantly concentrated on the description of cross-sectional patterns of multimorbidity, including prevalence and clusters of multimorbidity [10]. However, multimorbidity develops sequentially as individuals accumulate diagnoses. It is essential to understand the accumulation of disease over time and the association between potentially important factors and this accumulation process [10]. This may provide nuanced information that is critical for primary and secondary prevention of disease and patient-specific management at each disease stage [8, 11, 14, 15]. However, there is still a lack of research on the longitudinal progression of CRMM and effective interventions to delay or prevent this progression.

Modifiable lifestyle behaviors are regarded as the easiest way to prevent or slow down the progression of diseases [11]. Prior studies have either investigated the impact of lifestyle on the onset of a single CRMD in participants free of any CRMD [16, 17], or on the prognosis of patients with CRMD or CRMM [18], which makes understanding the impact of lifestyle on the entire longitudinal progression challenging. There is a lack of studies that estimate the relationships between lifestyle factors and longitudinal progression of CRMM. Furthermore, previous studies have only focused on a limited selection of conventional lifestyle factors, without considering the emerging factors (such as sleep, sedentary behavior, and social connection). Also, previous research has not considered the relative contribution of each factor. These could inform the development of effective lifestyle interventions and help prioritize interventions related to the progression of CRMM.

In line with previous studies [8, 10, 19–21], the progress of multimorbidity can be defined as the transitions of individuals through various comorbidity states during follow-up. Multistate models (MSMs) extend the traditional survival model to encompass more than two disease states, providing a useful framework for modelling complex longitudinal disease accumulation data. That has been widely used in studies on progression of multimorbidity [8, 10, 19–21]. To address above research gaps, we conducted a prospective MSM analyses to examine association between baseline lifestyle and the longitudinal progression of CRMM, based on the nearly 360,000 UK Biobank participants with a follow-up of approximately 14 years. Specifically, a series of questions were investigated. First, what is the role of lifestyle on the cumulative number of diseases over time, i.e., from healthy to FCRMD, subsequently to CRMM, and further to death? And what is the relative contribution of each lifestyle factor (including conventional and emerging factors)? Second, how does the role of lifestyle vary in all possible transitions when we further consider the specific types of FCRMD and CRMM?

## Methods

### Study design and participants

**Fig. 1:**
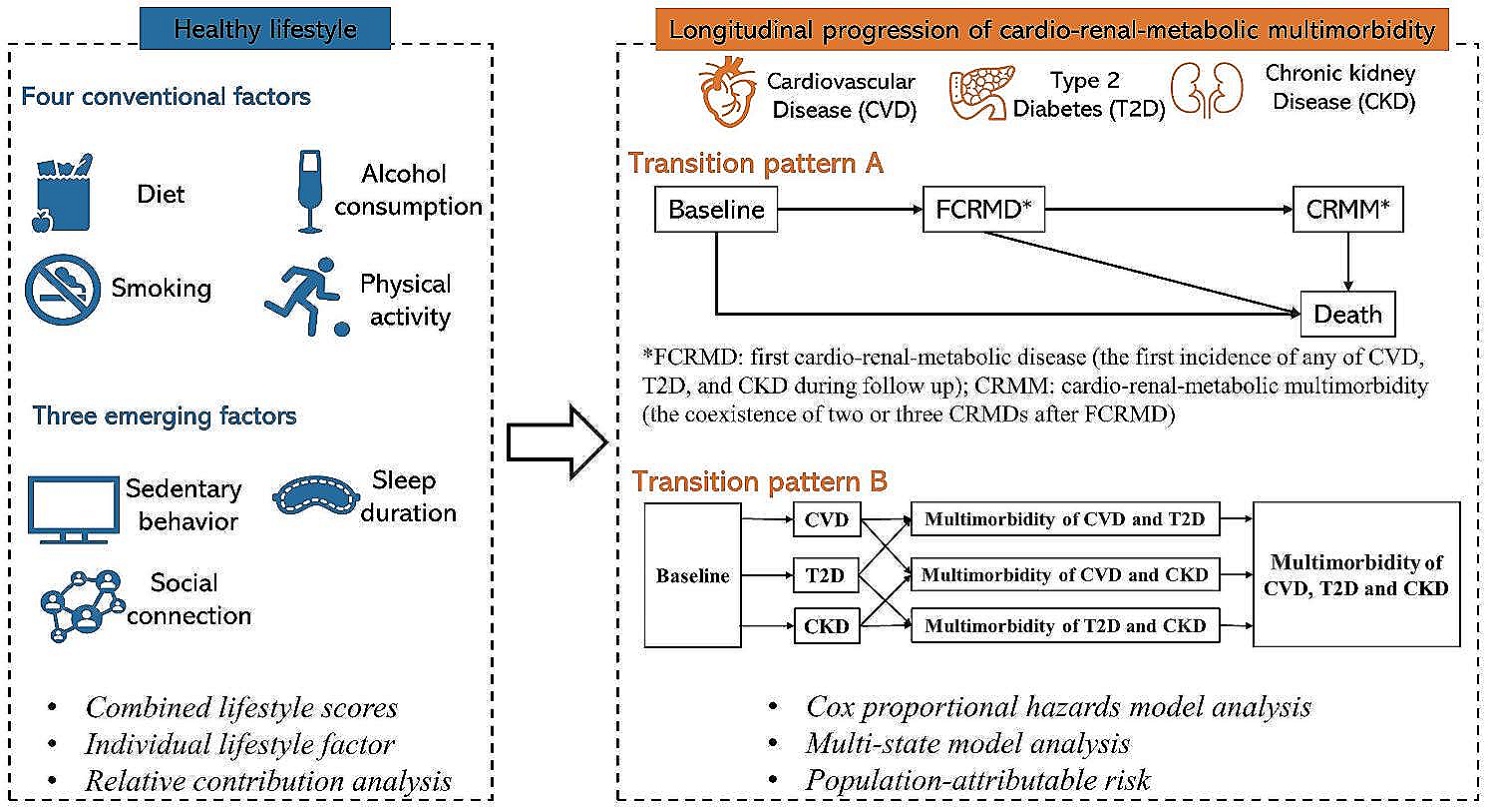
Flowchart of overall study design

The overall study design is shown in Fig. 1. FCRMD was defined as the first incidence of any of CVD, T2D, and CKD during follow up [1, 3]. CRMM was defined as the coexistence of two or three CRMDs after FCRMD [1]. We considered seven lifestyle factors, including diet, alcohol consumption, smoking, physical activity, sedentary behavior, sleep duration, and social connection. In this longitudinal cohort study, we first focus on the cumulative number of diseases over time, without considering the specific type, i.e., from healthy to FCRMD, then to CRMM and further to death (transition pattern A in Fig. 1). We estimated the associations of overall lifestyle score with disease progression in transition pattern A and assessed the relative contribution of each lifestyle factor in each transition. On the basis of transition pattern A, we further consider the specific types of FCRMD and CRMM in the progression, e.g., from healthy to CVD, then to comorbidity of CVD and T2D, to comorbidity of CVD, T2D and CKD (transition pattern B in Fig. 1).

Participants in this study were drawn from the UK Biobank, a prospective cohort study with over 500,000 participants. As detailed previously [22], participants aged 37 to 73 years underwent assessments between 2006 and 2010, including electronic questionnaires, physical examinations, and biochemical tests. The UK Biobank was approved by the North West Multicenter Research Ethical Committee. All participants provided written informed consent. Baseline questionnaire information was used to assess lifestyle. Longitudinal follow-up data based on the UK Biobank enabled us to observe the status and specific timing of the onset of CVD, T2D and CKD, as well as death. We assessed the transitions of longitudinal disease progression based on this information. The corresponding relationship between observed outcome data during follow up and transition pattern A and B of disease progression is detailed in online Appendix Figure S1.

For the current analysis, we excluded participants diagnosed with CVD, T2D or CKD before baseline (*n* = 68,975), participants with missing data on any lifestyle factor measurements (*n* = 70,562), and participants with missing data on select covariates (*n* = 5266). Finally, a total of 357,554 participants were included in the present analysis. See online Appendix Figure S2 for the selection flow chart of the participants. See online Appendix Table S1, Table S2 for missing information on each variable. This research has been conducted using the UK Biobank Resource under Application Number 117,185.

### Follow-up for cardio-renal-metabolic disease and death

FCRMD was defined as the first incidence of any of CVD, T2D, and CKD during follow up [1, 3]. CRMM was defined as the coexistence of two or three CRMDs after FCRMD [1]. In the UK Biobank, the incidence of CVD, T2D, CKD and death was obtained from the following sources: self-reported information, primary care data, and hospital admission data. Health data were available up to 31 October 2022, 31 August 2022, and 31 May 2022 for England, Scotland, and Wales, respectively (https://biobank.ndph.ox.ac.uk/showcase/exinfo.cgi? src=Data_providers_and_dates). Follow-up was censored at death, loss to follow-up, or the last date with available information, whichever came first. According to previous studies [2, 23], the diagnoses of CVD, T2D and CKD were ascertained according to the corresponding International Classification of Diseases, 10th Revision (ICD-10) or the Office of Population Censuses and Surveys Classification of Interventions and Procedures, version 4 (OPCS-4) codes. Incident CVD was defined using codes corresponding to coronary heart disease, atrial fibrillation, heart failure, peripheral artery disease and stroke. More details of outcomes ascertainment are shown in online Appendix Table S3.

### Assessment of lifestyle factors and other covariates

In line with previous studies [18, 24, 25], we constructed a lifestyle score with seven modifiable behavioral factors, measured at baseline using a touchscreen questionnaire. These factors included four conventional factors (i.e., diet, alcohol consumption, smoking, physical activity) and three emerging factors (i.e., sedentary behavior, sleep duration, and social connection). Participants received 1 point for each factor aligned with national guidelines and recommendations [26–30]. Higher lifestyle scores indicate better adherence to lifestyle recommendations. See online online Appendix Text S1 for detailed definitions of healthy lifestyle. Briefly, a low-risk diet was defined as an adequate intake of at least four of seven food groups recommended as dietary priorities: including fruits, vegetables, fish, processed meats, unprocessed red meats, whole grains, and refined grains. Low-risk alcohol consumption was defined as moderate drinking (0–14 g per day for women or 0–28 g per day for men) on a relatively regular frequency [25, 31]. Participants who reported no drinking or only drinking on special occasions were regarded as irregular drinkers. Never smoking was classified as low risk. Regular physical activity was defined as ≥ 150 min moderate activity per week, or ≥ 75 min vigorous activity per week, or an equivalent combination, or moderate activity ≥ 5 times/week, or vigorous activity ≥ 1 time/week. Low-to-moderate sedentary behavior (≤ 4 h per day watching TV and using a computer) was classified as low risk. Low-risk sleep was defined as 7–9 h of sleep duration. Low-risk social connection was defined as frequent social connection (≤ 1 on the social isolation index). Social isolation index was calculated based on the sum of the following three indices: the number in the household, frequency of friend/family visits, and participation in leisure/social activity [29]. The lifestyle scores were divided into three categories for additional analysis: positive (6–7 scores), intermediate (3–5), and unfavorable (0–2). Consistent with previous studies [18, 24, 25], body mass index (BMI) was not included in our lifestyle score, given the obesity paradox [32] and its possible influence on lifestyle. Nevertheless, we included BMI in the lifestyle score in the sensitivity analysis, with a low-risk level defined as a BMI of 18.5–25 kg/m^2^.

Regarding covariates, as previously reported [8, 33, 34], potential confounders included age (continuous, years), sex (male, female), race (white, non-white), Townsend deprivation index (continuous, a composite measure of deprivation based on four key variables [35]: unemployment, non-car ownership, non–home ownership, and household overcrowding), education (no qualification, any other qualifications, college or university degree), BMI (continuous, kg/m^2^), and assessment centers (England, Wales, Scotland).

### Statistical analyses

We used means (standard deviations) or frequencies (proportions) for continuous and categorical variables, respectively, to describe characteristics of total participants, participants with FCRMD, and participants with CRMM.

In the initial analysis, we first used the traditional Cox proportional hazards model to estimate the associations of individual and combined lifestyle scores with each disease states (i.e., FCRMD, CRMM, and all-cause death). The proportional hazard assumption was checked using the Schoenfeld residuals plots, and no significant violations were detected.

In main analyses, we first used MSMs [36] to assess the association between overall lifestyle score and longitudinal progression from healthy to incident FCRMD, then to CRMM, and ultimately to death (transition pattern A, Fig. 1). We then estimated the association of individual lifestyle factor with all transitions of CRMM progression and assessed the relative contributions. MSM extend the traditional survival model to encompass more than two disease states [8, 19–21]. There were five transitions between the three states: (1) baseline to FCRMD, (2) baseline to death from a disease other than CRMD, (3) FCRMD to CRMM, (4) FCRMD to death from any cause, (5) CRMM to death from any cause. For those participants entering different states on the same date, we calculated the entering date of the theoretical prior state as the entering date of the latter state minus 0.5 days. Two multivariable adjusted models were constructed: model 1 was adjusted for age and sex; model 2 was further adjusted for race, Townsend deprivation index, education, BMI, and assessment centers. For analyses on individual lifestyle factors, all lifestyle factors were simultaneously adjusted. To evaluate the relative proportion of individual lifestyle scores in each transition of CRMM progression, we constructed quantile G-computation [37] (QGC), which estimates positive or negative relative contribution of each component and has been widely used in epidemiological research [38, 39]. More details of QGC method are shown in online Appendix Text S2 The population-attributable risk (PAR) was applied to examine the proportion of each transition of CRMM that theoretically would not have occurred if all participants had adhered to 6–7 lifestyle factors. We calculated the PARs using the R package “AF” under the assumption of a causal relationship between lifestyle and disease progression [18, 40, 41]. Considering the PARs showed similar estimates from 1 to 10 years, we used time = 5 years for the analyses. We also predicted the transition probabilities over time from one state to another under unfavorable and positive lifestyle by using a MSM.

On the basis of transition pattern A, we further consider the specific types of FCRMD and CRMM in disease progression (transition pattern B, Fig. 1), the MSM consisted of seven states, including CVD, T2D, CKD, multimorbidity of CVD and T2D, multimorbidity of CVD and CKD, multimorbidity of T2D and CKD, and three CRMM. Accordingly, twelve transitions between seven states were considered. We excluded 2329 participants, leaving 355,225 participants in the analysis, because we could not ascertain the temporal sequence of disease occurrences if a participant was diagnosed with at least two of CVD, T2D or CKD on the same date.

To examine potential effect modifiers, the multistate analyses of transition pattern A were further stratified according to age (≤ 55 years or > 55 years), sex and Townsend deprivation index (≤ median or > median). Interactions were tested by using the likelihood ratio test, which compared models with and without a cross-product term.

Several sensitivity analyses for the multistate analyses of transition pattern A were conducted to test the robustness of our results. First, we constructed a lifestyle score with four conventional factors (i.e., diet, alcohol consumption, smoking, physical activity) and explored associations with the disease progression. Second, we included BMI in the lifestyle score. Low-risk level of BMI was defined as 18.5–25 kg/m^2^. Third, the low-risk level for alcohol consumption was redefined as no heavy drinking. Fourth, we further adjusted the self-reported overall health rating, family history of CVD or T2D and related drug information (medication for cholesterol, blood pressure or diabetes) to reduce the influence of poor health on lifestyle. Fifth, we further adjusted baseline blood pressure and lipids as covariates. Sixth, we excluded outcome events that occurred within the first six months of follow-up to examine the magnitude of potential reverse causality. Seventh, for those participants who entering different states on the same date, we calculated the entering date of the prior status using different time intervals (1, 30, and 365 days) to assess the impact of these intervals on the results. Eighth, the MSM also consisted of four states including FCRMD, two CRMM (the coexistence of two CRMD), three CRMM (the coexistence of two CRMD) and death, to explore associations with the longitudinal progression of CRMM in more detail. This transition pattern is shown in online Appendix Figure S3. Finally, we further divided the transition from the baseline to the incident FCRMD into three separate transitions (baseline to CVD, baseline to T2D, and baseline to CKD), to explore associations with specific diseases. This transition pattern is shown in online Appendix Figure S4. All analyses were conducted using R version 4.1.1.

## Results

### Characteristics of the participants

Among 357,554 participants, the mean age was 55.86 (SD 8.06) years, 158,824 (44.4%) participants were male, and 342,024 (95.7%) were White ethnicity. The characteristics of the total participants, those with FCRMD, and those with CRMM are shown in Table 1. Participants with CRM conditions were more likely to be older, male, have a lower education level and a higher Townsend deprivation index. In terms of lifestyle, participants with CRM conditions tended to have a lower overall lifestyle score and more like to smoke, have high-risk diet habits and sedentary behaviors.

**Table 1:** Baseline characteristics of study participants by incident disease states

Characteristics	Total (*N* = 357,554)	Participants with FCRMD (*N* = 69,238)	Participants with CRMM (*N* = 10,303)
Age at baseline, year	55.86 (8.06)	59.54 (7.15)	61.10 (6.61)
Male (%)	158,824 (44.4)	38,276 (55.3)	5934 (57.6)
White ethnicity (%)	342,024 (95.7)	66,323 (95.8)	9732 (94.5)
Education (%)			
No qualification	48,554 (13.6)	14,587 (21.1)	2917 (28.3)
Any other qualifications	180,010 (50.3)	34,780 (50.2)	5025 (48.8)
College or university degree	128,990 (36.1)	19,871 (28.7)	2361 (22.9)
BMI, kg/m^2^	26.95 (4.49)	28.32 (4.93)	29.86 (5.37)
Townsend deprivation index	− 1.51 (2.96)	− 1.33 (3.07)	− 1.01 (3.22)
Assessment centers			
England	318,266 (89.0)	62,430 (90.2)	9603 (93.2)
Wales	14,496 (4.1)	2706 (3.9)	278 (2.7)
Scotland	24,792 (6.9)	4102 (5.9)	422 (4.1)
Lifestyle class (%)			
Unfavorable [0–2]	26,438 (7.4)	7443 (10.7)	1482 (14.4)
Intermediate [1–3]	238,504 (66.7)	48,065 (69.4)	7312 (71.0)
Positive [4, 5]	92,612 (25.9)	13,730 (19.8)	1509 (14.6)
Overall lifestyle score	4.55 (1.37)	4.27 (1.40)	4.00 (1.40)
Lifestyle component (%)			
Low-risk diet	198,157 (55.4)	36,484 (52.7)	4974 (48.3)
Low-risk alcohol consumption	185,137 (51.8)	33,834 (48.9)	4761 (46.2)
Never smoking	200,481 (56.1)	33,619 (48.6)	4483 (43.5)
Regular physical activity	273,552 (76.5)	51,410 (74.3)	7274 (70.6)
Low-to-Moderate sedentary behavior	191,778 (53.6)	31,263 (45.2)	3959 (38.4)
Low-risk sleep	268,122 (75.0)	49,792 (71.9)	7166 (69.6)
Frequent social connection	309,792 (86.6)	59,444 (85.9)	8642 (83.9)

*FCRMD *first cardio-renal-metabolic disease, *CRMM *cardio-renal-metabolic multimorbidity (the coexistence of two or three CRMDs after FCRMD).

Data are expressed as mean (standard deviation) or numbers (percentage)

**Fig. 2:**
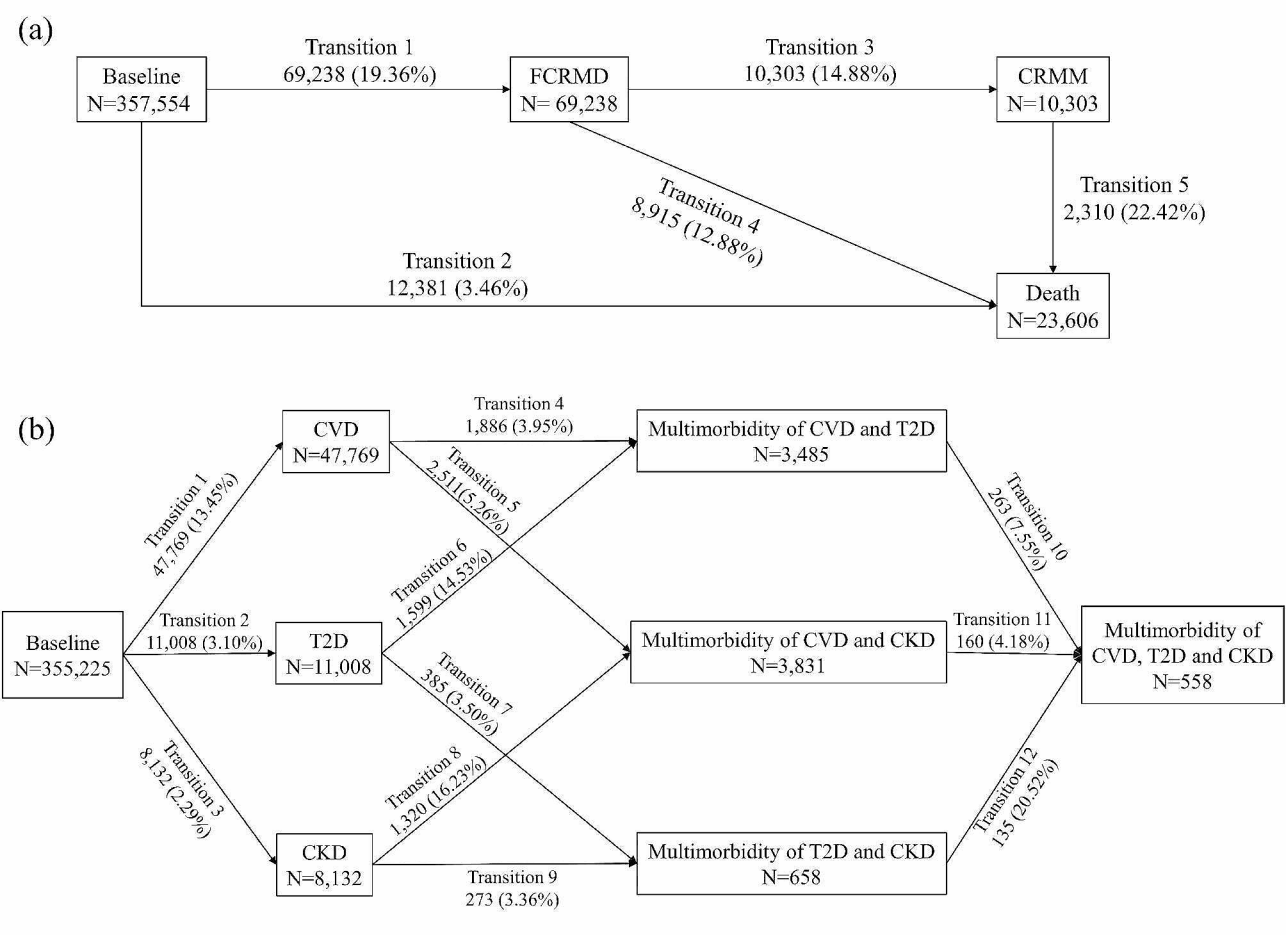
Numbers (percentages) of participants in longitudinal disease progression. **a** Numbers (percentages) of participants in transition pattern A from baseline to FCRMD, CRMM and death. **b** Numbers (percentages) of participants in transition pattern B from baseline to specific FCRMD, then to specific two CRMM, and ultimately to three CRMM. *FCRMD *first cardio-renal-metabolic disease, *CRMM *cardio-renal-metabolic multimorbidity (the coexistence of two or three CRMDs); *CVD *cardiovascular disease; *T2D *type 2 diabetes, *CKD *chronic kidney disease

The numbers (percentages) of participants in longitudinal disease progression are shown in Fig. 2. During a median follow-up of 13.62 (IQR: 12.90–14.29) years, 69,238 (19.36%) participants experienced FCRMD; 10,303 (14.88%) participants with FCRMD were developed CRMM; 2310 (22.42%) participants with CRMM died from any cause (Fig. 2a). Regarding the disease-specific transitions (2329 participants were excluded), there were 47,769 (13.45%) first incident cases of CVD, 11,008 (3.10%) first incident cases of T2D, and 8132 (2.29%) first incident cases of CKD (Fig. 2b).

### Lifestyle and longitudinal progression of CRMM in transition pattern A

We first examined the relationships of the individual and overall lifestyle scores with each disease state (i.e., FCRMD, CRMM, and death) using Cox regression models. Both individual and combined lifestyle scores were associated with the risks of FCRMD, CRMM, and death (online Appendix Table S4).

The MSMs analysis results of the associations of combined lifestyle scores with the progression of CRMM (transition pattern A) are shown in Table 2 (Model 2) and online Appendix Table S5 (Model 1). Model 1 and model 2 showed similar results. In short, a high lifestyle score was associated with the reduced risk of all transitions from healthy to FCRMD, to CRMM and to death. When all 7 lifestyle factors were combined, for the transition from baseline to FCRMD, the full adjusted hazard ratios (HRs) for participants scoring 3–5, and 6–7, as compared with those scoring 0–2, were 0.76 (95% CI 0.74, 0.78), and 0.65 (95% CI 0.63, 0.67), respectively. The HR of each 1-point increase was 0.91 (95% CI 0.90, 0.91). For the transition from FCRMD to CRMM, the full adjusted HRs of for participants scoring 3–5, and 6–7, as compared with those scoring 0–2, were 0.78 (95% CI 0.74, 0.82), and 0.64 (95% CI 0.60, 0.69), respectively. The HR of each 1-point increase was 0.90 (95% CI 0.89, 0.91). We found a statistically significant association between combined lifestyle scores and the transition from baseline to death, with the association being slightly stronger compared to the transitions from baseline to FCRMD and FCRMD to CRMM. For participants with relatively poor adherence (0–5) to a healthy lifestyle (6–7), the PARs for the transition from baseline to FCRMD, the transition from FCRMD to CRMM and the transition from baseline to death were 12.82% (95% CI 11.54%, 14.10%), 15.71% (95% CI 11.92%, 19.49%) and 19.60% (95% CI 16.73%, 22.47%), respectively. Our results showed that positive lifestyle was associated with a low transition probability to a more serious state, except for the transition from baseline to death (online Appendix Figure S5).

**Table 2:** Multistate model to assess associations of combined lifestyle scores with different transitions in transition pattern A among 357,554 participants

Over lifestyle score	Baseline to FCRMD		Baseline to Death		FCRMD to CRMM		FCRMD to Death		FCRMM to Death	
	HR (95% CI)	*P*	HR (95% CI)	*P*	HR (95% CI)	*P*	HR (95% CI)	*P*	HR (95% CI)	*P*
[0 ~ 2]	1		1		1		1		1	
[3 ~ 5]	0.76 (0.74, 0.78)	< 0.001	0.59 (0.55, 0.62)	< 0.001	0.78 (0.74, 0.82)	< 0.001	0.65 (0.61, 0.69)	< 0.001	0.79 (0.71, 0.89)	< 0.001
[6 ~ 7]	0.65 (0.63, 0.67)	< 0.001	0.47 (0.44, 0.50)	< 0.001	0.64 (0.60, 0.69)	< 0.001	0.55 (0.51, 0.60)	< 0.001	0.64 (0.55, 0.75)	< 0.001
Per score point	0.91 (0.90, 0.91)	< 0.001	0.84 (0.83, 0.86)	< 0.001	0.90 (0.89, 0.91)	< 0.001	0.87 (0.86, 0.89)	< 0.001	0.90 (0.88, 0.93)	< 0.001
PAR, %	12.82 (11.54, 14.10)	< 0.001	19.60 (16.73, 22.47)	< 0.001	15.71 (11.92, 19.49)	< 0.001	13.90 (10.11, 17.68)	< 0.001	15.70 (7.25, 24.15)	< 0.001

Transition pattern A was defined as transition from baseline to FCRMD, then to CRMM, and subsequently to death. Model was adjusted for age, sex, race, Townsend deprivation index, education, BMI, and assessment centers

*HR *hazard ratio, *CI *confidence interval, *FCRMD *first cardio-renal-metabolic disease, *CRMM *cardio-renal-metabolic multimorbidity (the coexistence of two or three CRMDs); *CVD *cardiovascular disease, *T2D *type 2 diabetes, *CKD *chronic kidney disease,* PAR *population-attributable risk

The results of the associations of individual lifestyle factors with the progression of CRMM (transition pattern A) are shown in Table 3 (Model 2) and online Appendix Table S5 (Model 1). The high scores of each lifestyle factor were associated with the decreased risk of all transitions from healthy to FCRMD, to CRMM and to death. For example, for the transition from baseline to FCRMD, the full adjusted HR for never smoking was 0.86 (0.84, 0.87). For the transition from FCRMD to CRMM, the full adjusted HR for low-risk diet was 0.85 (0.81, 0.88). For the transition from FCRMD to death, the full adjusted HR for frequent social connection was 0.75 (0.71, 0.79). Smoking status contributed to high proportions for all transitions of disease progression, accounting for 19.88-38.10%. High-risk diet contributed the largest proportion to the risk of transition from FCRMD to CRMM, accounting for 22.53%. Less-frequent social connection contributed the largest proportion to the risk of transition from FCRMD to death, accounting for 28.81%.

**Table 3:**
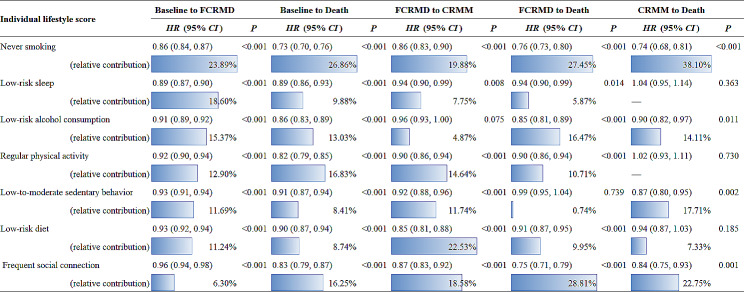
Multistate model to assess associations of individual lifestyle factor with different transitions in transition pattern A among 357,554 participants

### Lifestyle and disease-specific transitions in transition pattern B 

**Fig. 3:**
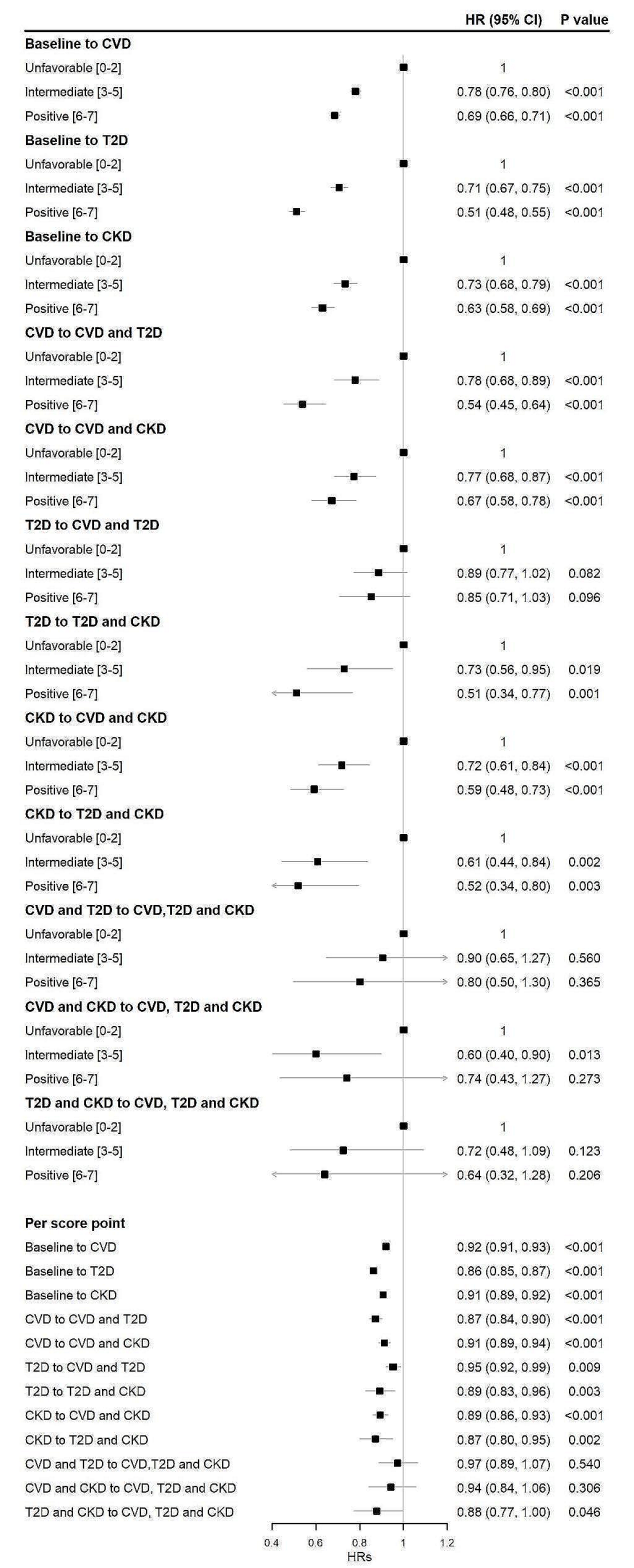
Multistate model to assess associations of lifestyle with different transitions in transition pattern B among 355,225 participants. *HR *hazard ratio; *CI *confidence interval; *CVD *cardiovascular disease; *T2D *type 2 diabetes; *CKD *chronic kidney disease. Transition pattern B was defined as transition from baseline to specific FCRMD, then to specific two CRMM, and ultimately to three CRMM. Model was adjusted for age, sex, race, Townsend deprivation index, education, BMI, and assessment centers

On the basis of transition pattern A, we further consider the specific types of FCRMD and CRMM in disease progression (transition pattern B), the results are shown in Fig. 3 (Model 2) and online Appendix Figure S6 (Model 1). In summary, overall lifestyle scores were associated with all transitions from healthy to specific FCRMD, then to specific two CRMM, and further to three CRMM, but to different extents. Compared to progression to CVD or CKD, lifestyle scores had slightly stronger associations with progression to T2D. The full adjusted HR for participants scoring 6–7, as compared with those scoring 0–2, was 0.51 (95% CI 0.48, 0.55) for the transition from baseline to T2D, 0.69 (95% CI 0.66, 0.71) for the transition from baseline to CVD, and 0.63 (95% CI 0.58, 0.69) for the transition from baseline to CKD, respectively. What’s more, the overall lifestyle scores were associated with the transitions from specific FCRMD to specific two CRMM. For example, the full adjusted HR for participants scoring 6–7, as compared with those scoring 0–2, were 0.54 (95% CI 0.45, 0.64) for the transition from CVD to multimorbidity of CVD and T2D, 0.67 (95% CI 0.58, 0.78) for the transition from CVD to multimorbidity of CVD and CKD, and 0.59 (95% CI 0.48, 0.73) for the transition from CKD to CVD and CKD, respectively. The overall lifestyle scores were associated with the transitions from specific two CRMM to three CRMM, although these associations were not statistically significant due to the sample size.

### Result of stratified and sensitivity analyses

Although several statistically significant interactions were found in the stratified analyses, most of them appeared clinically meaningless (online Appendix Table S6). The lifestyle scores showed stronger associations with the progression among the younger participants and low SES participants, compared to older participants and high SES participants. Sex modified the associations of overall lifestyle scores with transitions of disease progression, but with a different direction.

In sensitivity analyses, our results did not materially change when redefining lifestyle scores in various ways (online Appendix Tables S7–S9), when additionally adjusting for health-related variables or baseline blood pressure and lipids (online Appendix Tables S10, S11), when excluding outcome events that occurred within the first 6 months of follow-up (online Appendix Table S12), and when using different time intervals for participants entering different states on the same date (online Appendix Table S13). Moreover, our conclusions did not change when including four states (FRMD, two CRMM, three CRMM and death) in MSMs (online Appendix Table S14), and when dividing the transition from the baseline to the incident disease into three separate transitions (baseline to CVD, baseline to T2D, and baseline to CKD) in the MSMs (online Appendix Table S15).

## Discussion

Leveraging a prospective study of approximately 360,000 adults in the UK Biobank, this study was the first to evaluate the role of lifestyle factors (four conventional and three emerging factors) and their relative contributions in the longitudinal progression of CRMM. We found that individual and combined lifestyle scores were associated with the risk of all transitions from healthy to FCRMD, to CRMM, and then to death. Smoking status accounted for high proportions in the whole disease progress, and diet and social connection played important roles in specific transition of CRMM progression. Regarding the disease-specific transitions (transition pattern B), we found that lifestyle scores exhibited slightly stronger associations with progression to T2D than with progression to CVD or CKD.

Our study played a complementary role in understanding the longitudinal progression of CRMM. CVD, T2D, CKD and their multimorbidity have become urgent health problems of concern to AHA, the American Diabetes Association (ADA) and European Society of Cardiology (ESC) [12, 13, 42]. However, few studies have focused on the incidence, progression to multimorbidity and death of CRMD. A cross-sectional study based on the US adults assessed the prevalence and overlap of CRM condition and showed an increasing trend in CRMM [1]. Existing studies have evaluated multimorbidity only in patients at a specific disease stage [18, 43]. In our cohort population, after more than 13 years of follow-up, about 20% of healthy participants developed FCRMD, with CVD being the most prevalent. After FCRMD, 15% of participants with FCRMD developed to CRMM (see Fig. 2). This result was lower than previously reported [3], probably because of insufficiently follow-up times. Consistent with previous studies [42, 43], we found that CRMM patients had higher mortality rates than patients with FCRMD (22.42% vs. 12.88%). Given the serious consequences of CRM conditions and the current state of under-treatment [1, 7, 42, 43], the present study focused on the longitudinal progression of CRMM, which can inform more effective management and treatment.

The present study, for the first time, found that lifestyle plays a crucial role in the longitudinal progression of CRMM. Our findings align with and reinforce established evidence of studies, which have concentrated solely on the association between lifestyle and a specific disease state, such as onset of a specific CRMD [16, 44, 45], or the mortality among patients with CRMDs [18]. More importantly, our multistate analysis offers novel insights by revealing a detailed association between lifestyle and disease progression of CRMM. We found that a high baseline lifestyle score was associated with a reduced risk of FCRMD during follow-up, which may inform primary prevention in participants free of CRMD. Beyond these, we found that a high baseline lifestyle score was associated with a reduced risk of CRMM after FCRMD during follow-up. And our findings suggest a strong protective effect of healthy lifestyle on the risk of death after FCRMD and CRMM during follow-up. This highlights the value of secondary prevention in patients with FCMD and higher lifestyle score to reduce the additional risk of subsequent CRMM or death.

Regarding the disease-specific transitions (transition pattern B), our findings indicate that healthy lifestyle may produce protective effect at all possible transitions from healthy to specific FCRMD, specific two CRMM, and three CRMM. Our results suggest a slightly stronger association with developing to T2D compared to transition to CVD or CKD. Previous studies focusing on cardiometabolic multimorbidity [8] and multimorbidity of cancer and cardiometabolic diseases [19] have also found that a stronger association with developing to T2D compared to with other transitions. This association may be related to the fact that healthy lifestyle behaviors such as diet and exercise, have a direct effect on insulin sensitivity, which is a key determinant in the development of T2D [46, 47]. Our results suggest lifestyle interventions have the potential to prevent the progress of different types of CRM conditions, particularly T2D.

Previous studies have covered four conventional lifestyle factors (i.e., diet, alcohol consumption, smoking, physical activity), with rarely consideration of emerging factors (i.e., sedentary behavior, sleep duration, and social connection) [8, 18–20]. Our study supports previous study [48] by showing a stronger association between smoking status and whole disease progression compared with other factors, suggesting that tobacco control may be a prime target for intervention. Previous studies have shown that smoking can lead to chronic inflammation, endothelial dysfunction, and other CRM-related pathway abnormalities [49–51]. In the transition from FCRMD to CRMM, dietary factor accounted for the largest proportion (22.53%) of all lifestyle factors. Consistent with our results, a prospective study has shown a significant association between diet and cardiometabolic multimorbidity, which may be related to high seafood and polyunsaturated fatty acids intake [52]. Additionally, existing studies showed that social isolation is associated with high risk of a specific CRMD and mortality [53–55], and the present study adds to existing evidence highlights the potentially large impact of social connectedness on death after FCRMD. In summary, our study identified independent associations of both conventional and emerging lifestyle factors with progression of CRMM. Exploring emerging lifestyle factors may further contribute to preventing, controlling, or delaying the disease progression.

### Strengths and limitations

This study has several strengths. First and most notably, the present study estimated, for the first time, the relationship between lifestyle (including four conventional factors and three emerging factors) and longitudinal progression of CRMM using the MSMs. Second, the contribution of each lifestyle factor to the disease progression was estimated to inform the prioritization of interventions. Third, we also comprehensively evaluate the role of lifestyle on the progression of specific CRM types.

Nonetheless, limitations are worth noting. First, for participants entering different states on the same date, we used an interval of 0.5 days to calculate the onset date of disease, which may lead to inaccurate estimations. However, we used several different time intervals in a sensitivity analysis, and these analyses did not significantly change our results. Second, we did not have detailed drug information for participants with CRMDs, which may reduce the risk of subsequent disease progression. However, considering the accessibility of the UK Biobank data, we further adjusted the self-reported overall health rating, related drug information (medication for cholesterol, blood pressure or diabetes) and family history of CVD and T2D in a sensitivity analysis, and these analyses did not significantly change our results. Third, UK Biobank measured lifestyle factors of all participants only at baseline and could not capture the possible lifestyle changes during follow-up. Our use of baseline lifestyle measures may also help to avoid reverse causality due to lifestyle changes after a disease episode. Fourth, the participants in the UK Biobank were primarily Caucasians. And since the UK Biobank comprises volunteers, its participants may skew towards individuals with better health compared to the general population. Caution is needed when generalizing our findings to other populations. Fifth, the age range of participants in the UK Biobank was 37 to 73 years. We may not be able to get at the risk of progression of CRMM in people aged 74 and over. Caution is needed when generalizing our findings. Finally, despite reasonable control for confounders, the presence of residual confounding cannot be entirely ruled out.

## Conclusion

In conclusion, the present study indicates that healthy lifestyle may produce a protective effect throughout the longitudinal progression from healthy to FCRMD, to CRMM, and eventually to death. This informs the primary and secondary prevention of CRM conditions. Regarding individual factors, smoking status, diet, and social connection played pivotal roles in specific disease transitions. Our study also suggests lifestyle interventions have preventive potential for disease-specific transitions, especially for T2D.

### Supplementary Information

Supplementary Material 1.

## Data Availability

No datasets were generated or analysed during the current study.
