# Supplementary material for: Lifestyle factors and their relative contributions to longitudinal progression of cardio-renal-metabolic multimorbidity: a prospective cohort study
Source: Cardiovasc Diabetol. 2024 Jul 18;23:265. doi: 10.1186/s12933-024-02347-3 (PMC11264843; doi:10.1186/s12933-024-02347-3)
Supplement: Supplementary file 1 — Supplementary Material 1. [file 12933_2024_2347_MOESM1_ESM.docx]

**Appendix**

**Contents**

[Supplementary method 4](#_Toc170730312)

[**Text S1. The details of the lifestyle assessment** 4](#_Toc170730313)

[**Text S2. Technical details of quantile G-computation** 7](#_Toc170730314)

[Supplementary figures 8](#_Toc170730315)

[**Figure S1. The corresponding relationship of observational outcome data with longitudinal disease progression.** 8](#_Toc170730316)

[**Figure S2. The selection flow chart of the participants in this study.** 9](#_Toc170730317)

[**Figure S3. Numbers (percentages) of participants from baseline to FCRMD, two CRMM, three CRMM, and ultimately to death.** 10](#_Toc170730318)

[**Figure S4. Numbers (percentages) of participants from baseline to specific FCRMD, then to CRMM, and ultimately to death.** 11](#_Toc170730319)

[**Figure S5. The transition probabilities over time from baseline to FCRMD and death, from FCRMD to CRMM and death and from CRMM to death in unfavorable and positive lifestyle by using a multi-state model.** 12](#_Toc170730320)

[**Figure S6. The role of lifestyle in disease-specific transitions.** 13](#_Toc170730321)

[Supplementary tables 14](#_Toc170730322)

[**Table S1. Missing information on lifestyles.** 14](#_Toc170730323)

[**Table S2. Missing information on covariates.** 14](#_Toc170730324)

[**Table S3. International Classification of Disease and procedure codes used to ascertain incident outcomes.** 15](#_Toc170730325)

[**Table S4. Associations of lifestyle with the risks of first cardio-renal-metabolic disease, cardio-renal-metabolic multimorbidity, and death using the Cox model.** 16](#_Toc170730326)

[**Table S5. Associations of lifestyle with the risks of FCRMD, CRMM, and death using multi-state model.** 18](#_Toc170730327)

[**Table S6. The results of stratified analyses.** 19](#_Toc170730328)

[**Table S7. Association of lifestyle score with the disease progression, when constructing lifestyle score with four conventional factors.** 20](#_Toc170730329)

[**Table S8. Association of lifestyle score with the disease progression, when including body mass index in the lifestyle score.** 20](#_Toc170730330)

[**Table S9. Association of lifestyle score with the disease progression, redefining low-risk alcohol consumption as no heavy drinking.** 21](#_Toc170730331)

[**Table S10. Association of lifestyle score with the disease progression, when additionally adjusting for health-related variables.** 21](#_Toc170730332)

[**Table S11. Association of lifestyle score with the disease dynamic progression, when additionally adjusting for baseline systolic blood pressure, diastolic blood pressure and cholesterol.** 22](#_Toc170730333)

[**Table S12. Association of lifestyle score with the disease progression, excluding outcome events that occurred in the first six months of follow-up.** 22](#_Toc170730334)

[**Table S13. Association of lifestyle score with the disease progression, using different intervals for the participants entering different states on the same date.** 23](#_Toc170730335)

[**Table S14. Association of lifestyle score with progression from baseline to FCRMD, two CRMM, three CRMM, and ultimately to death.** 24](#_Toc170730336)

[**Table S15. Association of lifestyle score with progression from baseline to one of CVD, T2D, CKD, then to cardio-renal-metabolic multimorbidity (CRMM), and ultimately to death.** 26](#_Toc170730337)

[References 28](#_Toc170730338)

Supplementary method

**Text S1.** **The details of the lifestyle assessment**

As mentioned in an earlier study^1-3^, lifestyle included the following seven factors: diet, alcohol consumption, smoking, physical activity, sedentary behavior, sleep duration, and social connection. All information on lifestyle factors was measured at baseline using a touchscreen questionnaire. Detailed questionnaires could be obtained through the UK Biobank website (<https://biobank.ndph.ox.ac.uk/showcase/>). For each factor, participants scored 1 point for a category recommended in the national guidelines and recommendations. Detailed information can be obtained from the following table.

| **Lifestyle factor^1-3^** | **Details** | **Definition** | **Field ID** |
| --- | --- | --- | --- |
| Alcohol consumption | Followed the Dietary Guidelines for Americans (DGA)^4^, alcohol consumption may have beneficial effects when consumed in moderation - up to one drink/day for women and two drinks/day for men.  1 drink equivalent described as containing 14g of pure alcohol.  125ml wine=0.85 drink-equivalents,  4% ABV pint beer = 1.28 drink-equivalents,  25ml spirits=0.57 drink-equivalents,  50ml fortified wine= 0.56 drink-equivalents | Moderate alcohol consumption was defined as:  women: >0 and ≤14g/day,  men: >0 and ≤28g/day.  Moderate alcohol consumption was scored as 1. | 20117, 1558,  1568, 1578,  1588, 1598,  1608, 4407,  4418, 4429,  4440, 4451 |
| Diet | Followed the DGA^4^, low-risk diet was defined as an adequate intake of at least four of seven food groups recommended as dietary priorities.  1. Fruits: ≥ 3 servings/day  2. Vegetables: ≥ 3 servings/day  3. Fish: ≥ 2 servings/week  4. Processed meats: ≤ 1 serving/week  5. Unprocessed red meats: ≤ 1.5  servings/week  6. Whole grains: ≥ 3 servings/day  7. Refined grains: ≤1.5 servings/day | A healthy dietary pattern was defined as including at least four out of seven foods.  A healthy dietary pattern was scored as 1. | 1309, 1319,  1289, 1299,  1329, 1339,  1349, 1369,  1379, 1389,  1438, 1448,  1458, 1468 |
| Physical activity | According to World Health Organization guidelines on physical activity recommendation^5^, adults should do ≥150 minutes of moderate activity per week OR ≥ 75 minutes of vigorous activity per week OR an equivalent combination OR moderate physical activity at least 5 days a week or vigorous activity once a week (at least 10 minutes). | Meeting the criteria for healthy physical activity was scored as 1. | 884, 894,  904, 914 |
| Sleep | According to NHS, American Academy of Sleep Medicine (AASM), and Sleep Research Society (SRS) recommendation^6^, sleep duration of 7 to 9 hours was classified into the healthy lifestyle category. | Meeting the standard of healthy sleep is defined as 1. | 1160 |
| Smoking | Many UK national guidelines suggest quitting smoking, such as NHS, National Institute for Health and Care Excellence^7^, participants with never smoking status were classified as the healthy lifestyle category | Never smoking was scored as 1. | 20116 |
| Sedentary behavior | According to WHO guidelines on sedentary behavior recommendations^5^, sedentary behavior was measured by the sum of self-reported sedentary behavior hours spent watching TV and using a computer (do not include using a computer at work) during a typical day. Values greater than 24 hours per day were excluded. | Sedentary for less than 4 hours was scored as 1. | 1070, 1080 |
| Social  connection | The social connection was assessed according to the social isolation index^3,8^. The social isolation index was calculated based on the sum of the following three indices: the number in the household, frequency of friend/family visits, and participation in leisure/social activity^8^.  1. The least isolated: social isolation index was 0,  2. moderately isolated: social isolation index was 1,  3. The most isolated: social isolation index was 2 or 3. | The least and moderately isolated were defined as frequent social connections.  Frequent social connection was scored as 1. | 709, 1031, 6160 |

**Text S2. Technical details of quantile G-computation**

We used quantile G-computation (QGC)^9^ to estimate the relative contribution of a single lifestyle factor. The QGC obtains causal relationships and estimates positive or negative relative contribution for each component, which has been widely used in epidemiological research^10,11^. In our study, the QGC method was carried out through the following steps:

Step 1: Arrangement of component data. The component data could keep the original scale, or be converted into categorized coded data as required, such as in quartiles. For the convenience of explanation, we directly used scores (0 or 1) for each lifestyle factor in the current study.

Step 2: Fitting regression models. The required covariates could be included in the model, which was omitted here for the sake of concise symbolic expression. The model was as follows:

$$Y=\beta_{0}+\sum_{j=1}^{k} \beta_{j}X_{j}^{q}+\epsilon$$

In the current study, $k$ represented the total number of lifestyle components, and $\epsilon$ represented the residual term. $x_{j}^{q}$ was the score of $j$th lifestyle component. $\sum_{j}^{k} \beta_{j}$ was the mixture effect of the total lifestyles. Weight of component or relative contribution was defined as ${\beta_{j}}/{\sum_{j}^{k} \beta_{j}}$. It could be interpreted as the contribution of the $j$th component to the total effect when all the lifestyle components change from unhealthy to healthy at the same time. Given the overall lifestyle effect the weights are considered fixed and so do not have confidence intervals or p-values^12^. Because the estimation process of the above model did not limit the positive or negative of $\beta_{j}$, we could estimate the positive or negative weight of each component at the same time. When there were both positive and negative associations between components and outcome, the positive and negative weights were calculated separately, with all positive weights summing to 1 and all negative weights summing to 1.

Supplementary figures


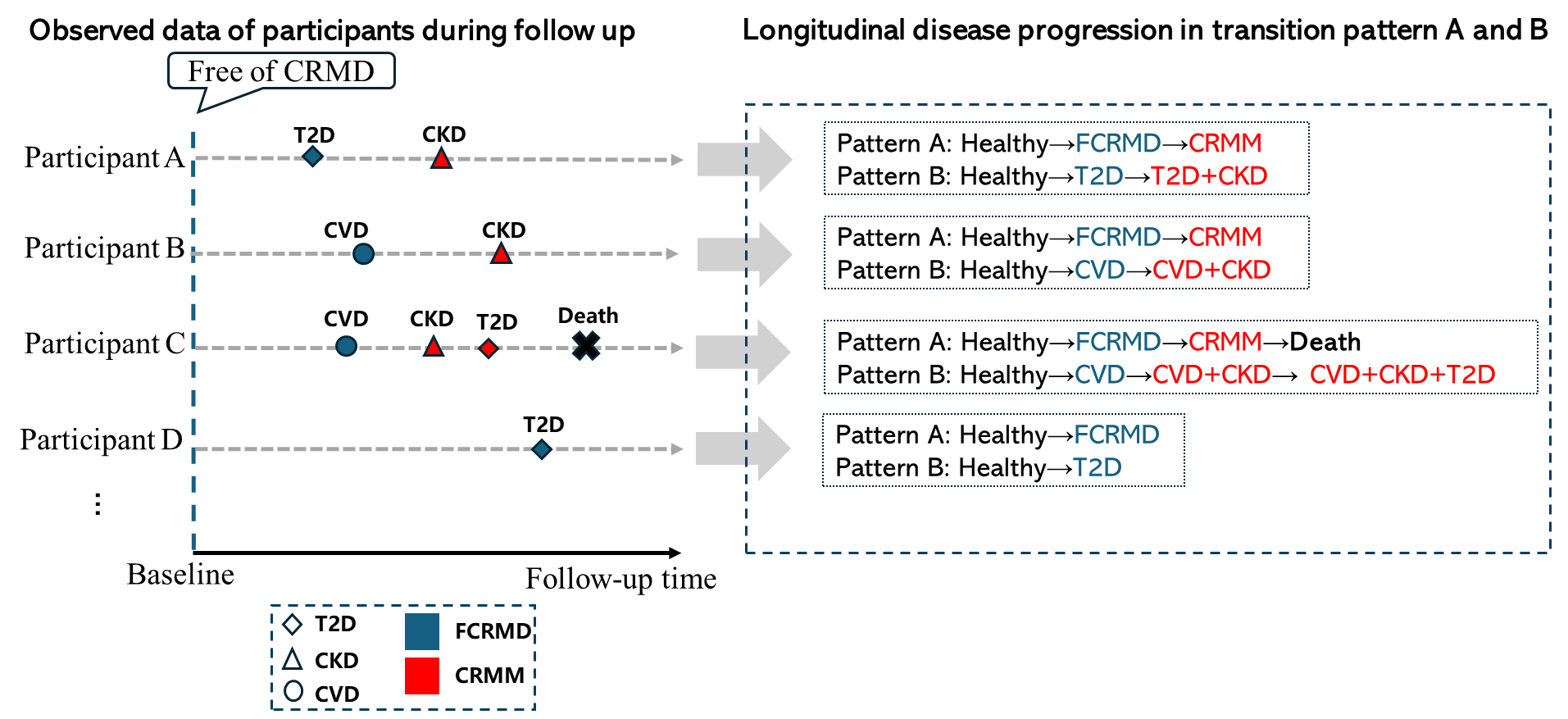


**Figure S1. The corresponding relationship of observational outcome data with longitudinal disease progression.**

Note: FCRMD: first cardio-renal-metabolic disease; CRMM: cardio-renal-metabolic multimorbidity (the coexistence of two or three CRMDs after FCRMD); CVD: cardiovascular disease; T2D: type 2 diabetes; CKD: chronic kidney disease. Transition pattern A was defined as transition from baseline to FCRMD, then to CRMM, and subsequently to death. Transition pattern B was defined as transition from baseline to specific FCRMD, then to specific two CRMM, and ultimately to three CRMM.


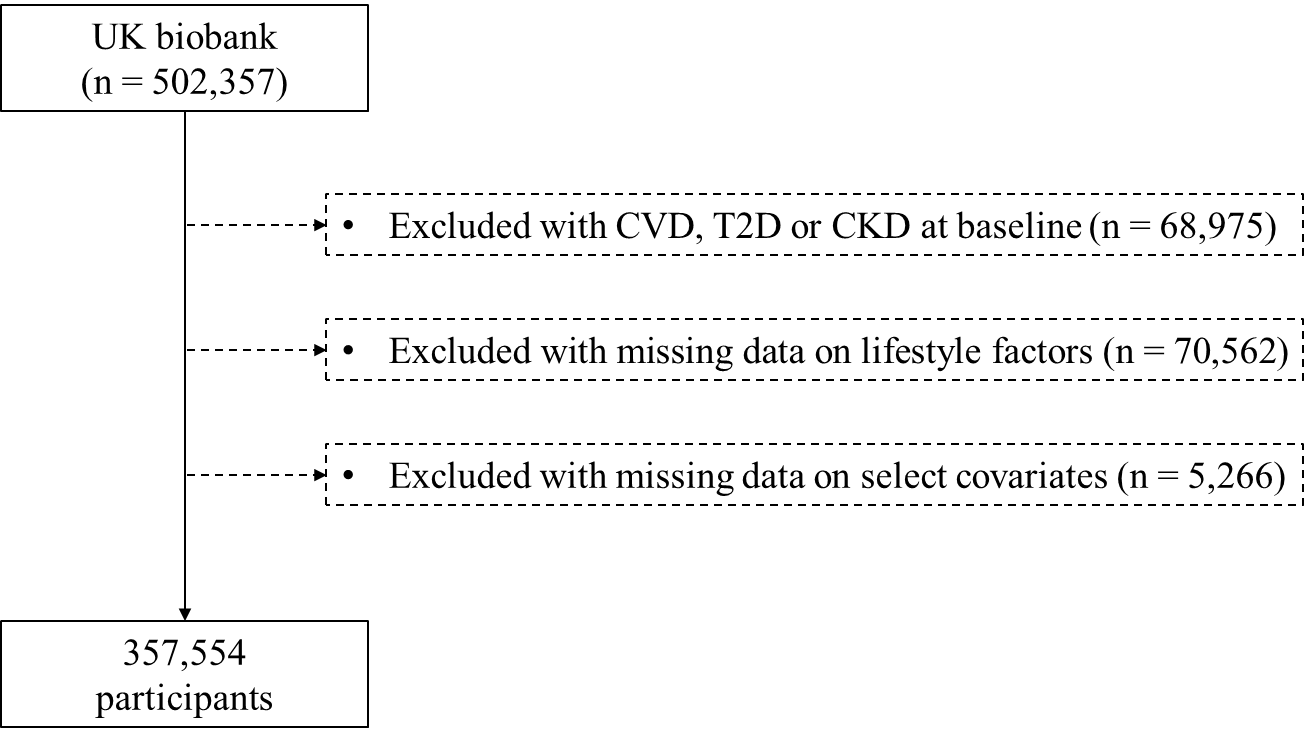


**Figure S2. The selection flow chart of the participants in this study.**

Note: CVD: cardiovascular disease, T2D: type 2 diabetes, CKD: chronic kidney disease.


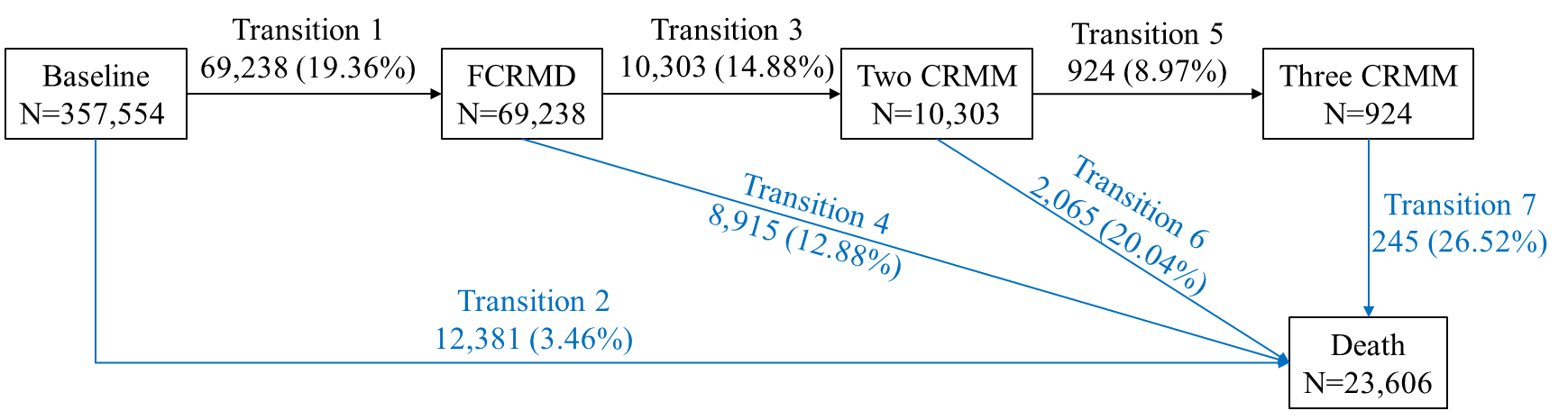


**Figure S3. Numbers (percentages) of participants from baseline to FCRMD, two CRMM, three CRMM, and ultimately to death.**

Note: FCRMD: first cardio-renal-metabolic disease; CRMM: cardio-renal-metabolic multimorbidity (the coexistence of two or three CRMDs after FCRMD). CVD: cardiovascular disease; T2D: type 2 diabetes; CKD: chronic kidney disease.


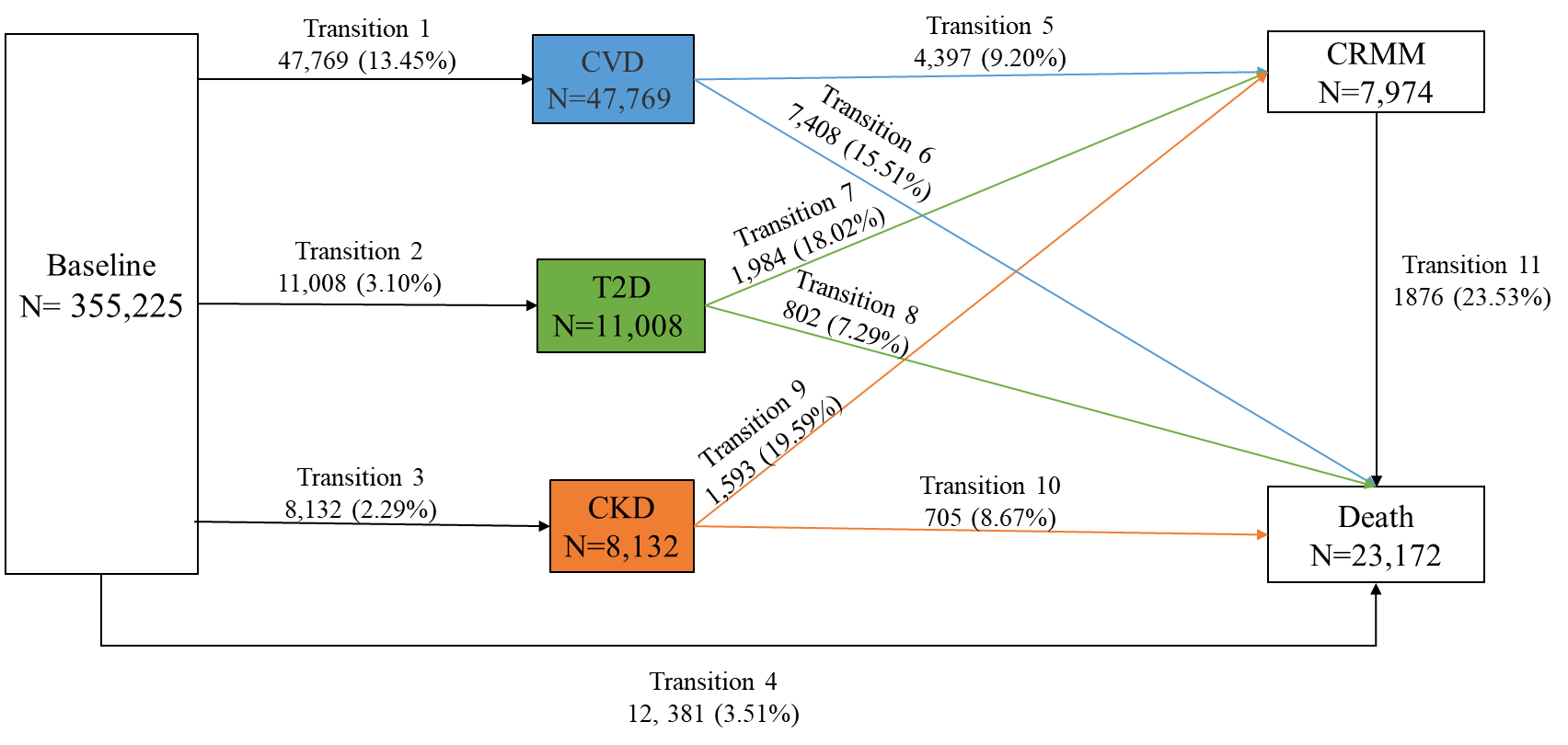


**Figure S4. Numbers (percentages) of participants from baseline to specific FCRMD, then to CRMM, and ultimately to death.**

Note: CVD: cardiovascular disease, T2D: type 2 diabetes, CKD: chronic kidney disease. CRMM: cardio-renal-metabolic multimorbidity (the coexistence of two or three CRMDs after FCRMD). We excluded 2329 participants, leaving 355,225 participants in the analysis, because we could not ascertain the temporal sequence of disease occurrences if a participant was diagnosed with at least two of CVD, T2D or CKD on the same date.


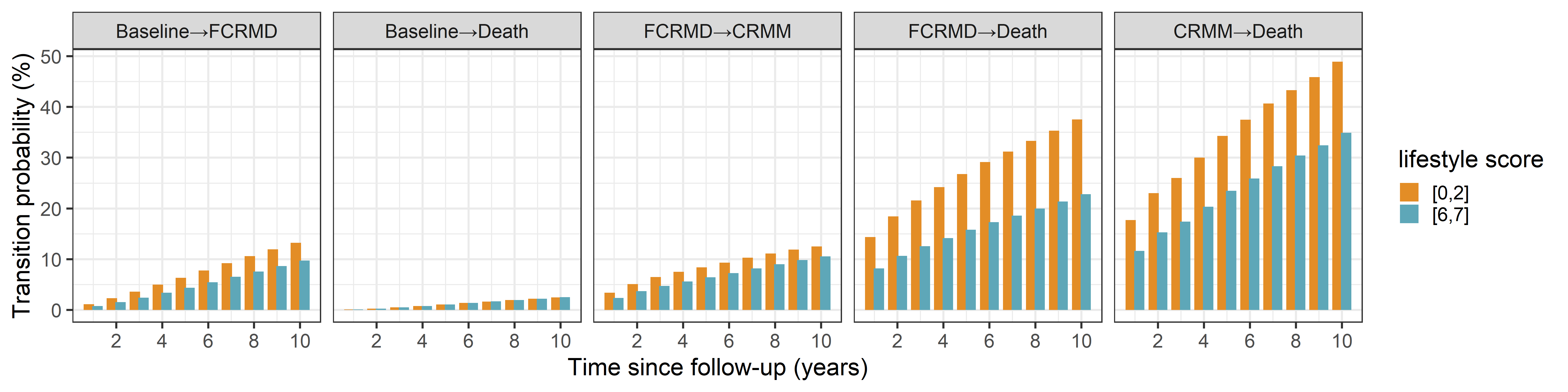


**Figure S5. The transition probabilities over time from baseline to FCRMD and death, from FCRMD to CRMM and death and from CRMM to death in unfavorable and positive lifestyle by using a multi-state model.**

Note: FCRMD: first cardio-renal-metabolic disease; CRMM: cardio-renal-metabolic multimorbidity (the coexistence of two or three CRMDs after FCRMD).


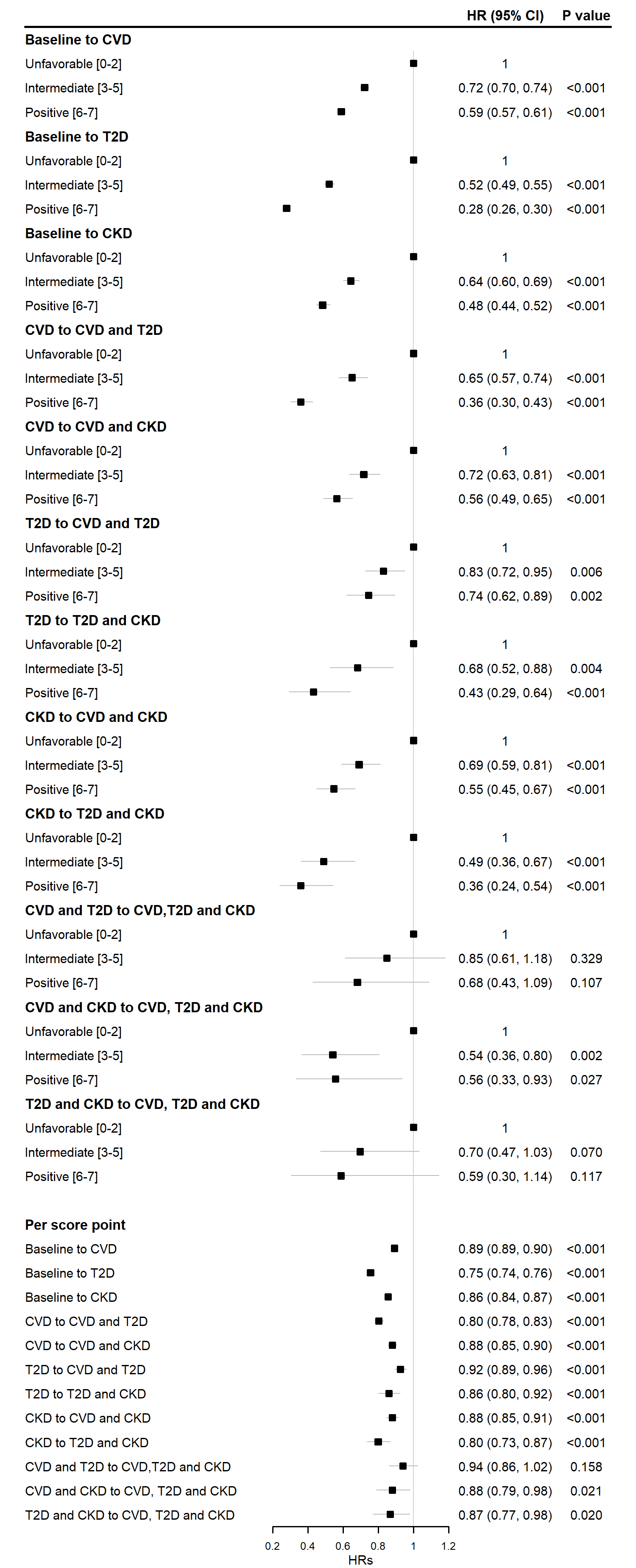


**Figure S6. The role of lifestyle in disease-specific transitions.**

Note: HR: hazard ratio, CI: confidence interval, CVD: cardiovascular disease, T2D: type 2 diabetes, CKD: chronic kidney disease. Model was adjusted for age and sex.

Supplementary tables

**Table S1. Missing information on lifestyles.**

| Characteristic | UK Biobank (n = 433382) |
| --- | --- |
| Diet | 10002 |
| Alcohol consumption | 38288 |
| Smoking | 2319 |
| Physical activity | 21285 |
| Sedentary behavior | 8155 |
| Sleep duration | 3297 |
| Social connection | 10270 |
| Combinations | 70562 |

Notes: Sample size of UK Biobank participants after exclusion of CVD, T2D or CKD at baseline.

**Table S2. Missing information on covariates.**

| Characteristic | UK biobank (n = 362820) |
| --- | --- |
| Age | 0 |
| Sex | 0 |
| Race | 1319 |
| Townsend deprivation index | 456 |
| Education | 2059 |
| BMI | 1509 |
| Assessment centers | 0 |
| Combinations | 5266 |

Notes: Sample size of UK Biobank participants after exclusion of CVD, T2D or CKD at baseline, and missing data on lifestyle factors

**Table S3.** **International Classification of Disease and procedure codes used to ascertain incident outcomes.**

| **Outcome^13^** | **ICD or procedural codes** |
| --- | --- |
| CVD^14^ | ICD-10:  I11.0, I20, I20.0, I20.1, I20.8, I20.9, I21, I21.0, I21.1, I21.2, I21.3, I21.4, I21.9, I22, I22.0, I22.1, I22.8, I22.9, I23, I23.0, I23.1, I23.2, I23.3, I23.4, I23.5, I23.6, I23.8, I24, I24.0, I24.1, I24.8, I24.9, I25, I25.0, I25.1, I25.2, I25.5, I25.6, I25.8, I25.9, I42.0, I42.1, I42.5, I42.8, I42.9, I48, I49, I49.0, I49.1, I49.2, I49.3, I49.4, I49.5, I49.8, I49.9, I50, I50.0, I50.1, I50.9, I70.0, I70.00, I70.01, 170.2, 170.20, I70.21, I70.8, I70.80, I70.9, I70.90, I73.8, I73.9  OPCS-4:  K40, K40.1, K40.2, K40.3, K40.4, K40.8, K40.9, K41, K41.1, K41.2, K41.3, K41.4, K41.8, K41.9, K42, K42.1, K42.2, K42.3, K42.4, K42.8, K42.9, K43, K43.1, K43.2, K43.3, K43.4, K43.8, K43.9, K44, K44.1, K44.2, K44.8, K44.9, K45, K45.1, K45.2, K45.3, K45.4, K45.5, K45.6, K45.8, K45.9, K46, K46.1, K46.2, K46.3, K46.4, K46.5, K46.8, K46.9, K47, K47.1, K47.2, K47.3, K47.4, K47.5, K47.8, K47.9, K48.1, K48.2, K48.3, K48.4, K48.8, K48.9, K49, K49.1, K49.2, K49.3, K49.4, K49.8, K49.9, K50, K50.1, K50.2, K50.3, K50.4, K50.8, K50.9, K57.1, K62.1, K62.2, K62.3, K62.4, K63, K75, X09.3, X09.4, X09.5, L21.6, L51.3, L51.6, L51.8, L52.1, L52.2, L54.1, L54.4, L54.8, L59.1, L59.2, L59.3, L59.4, L59.5, L59.6, L59.7, L59.8, L60.1, L60.2, L63.1, L63.5, L63.9, L66.7, X50.1, X50.2 |
| T2D^15^ | ICD-10: E11, E14 |
| CKD^14^ | ICD-10: N18, N18.0, N18.1, N18.2, N18.3, N18.4, N18.5, N18.8, N18.9  OPCS-4: M01, M01.1, M01.2, M01.3, M01.4, M01.5, M01.8, M01.9 |

Note: CVD: cardiovascular disease, T2D: type 2 diabetes, CKD: chronic kidney disease, ICD: International Classification of Disease, OPCS-4: Office of Population Censuses and Surveys Classification of Interventions and Procedures, version 4.

**Table S4. Associations of lifestyle with the risks of first cardio-renal-metabolic disease, cardio-renal-metabolic multimorbidity, and death using the Cox model.**

|  | **FCRMD** | | **CRMM** | | **Death** | |
| --- | --- | --- | --- | --- | --- | --- |
|  | **Model 1** | **Model 2** | **Model 1** | **Model 2** | **Model 1** | **Model 2** |
| **Over lifestyle score** |  |  |  |  |  |  |
| [0~2] | 1 | 1 | 1 | 1 | 1 | 1 |
| [3~5] | 0.66 (0.65, 0.68) | 0.76 (0.74, 0.78) | 0.51 (0.48, 0.54) | 0.65 (0.62, 0.69) | 0.52 (0.50, 0.54) | 0.57 (0.55, 0.59) |
| [6~7] | 0.50 (0.49, 0.52) | 0.65 (0.63, 0.67) | 0.29 (0.27, 0.31) | 0.47 (0.44, 0.51) | 0.38 (0.36, 0.40) | 0.44 (0.42, 0.46) |
| Per score point | 0.86 (0.85, 0.86) | 0.91 (0.90, 0.91) | 0.76 (0.74, 0.77) | 0.84 (0.83, 0.85) | 0.80 (0.80, 0.81) | 0.83 (0.82, 0.84) |
| **Individual lifestyle score** |  |  |  |  |  |  |
| Low-risk diet | 0.90 (0.88, 0.91) | 0.93 (0.92, 0.94) | 0.76 (0.73, 0.79) | 0.81 (0.78, 0.85) | 0.85 (0.83, 0.88) | 0.88 (0.85, 0.90) |
| Low-risk alcohol consumption | 0.86 (0.84, 0.87) | 0.91 (0.89, 0.92) | 0.80 (0.76, 0.83) | 0.89 (0.85, 0.92) | 0.80 (0.78, 0.82) | 0.83 (0.81, 0.85) |
| Never smoking | 0.83 (0.82, 0.84) | 0.86 (0.84, 0.87) | 0.74 (0.71, 0.77) | 0.78 (0.75, 0.81) | 0.68 (0.66, 0.69) | 0.70 (0.68, 0.72) |
| Regular physical activity | 0.87 (0.86, 0.89) | 0.92 (0.90, 0.94) | 0.76 (0.73, 0.79) | 0.84 (0.81, 0.88) | 0.83 (0.80, 0.85) | 0.83 (0.81, 0.86) |
| Low-to-moderate sedentary behavior | 0.83 (0.81, 0.84) | 0.93 (0.91, 0.94) | 0.70 (0.67, 0.72) | 0.86 (0.82, 0.89) | 0.88 (0.85, 0.90) | 0.91 (0.89, 0.94) |
| Low-risk sleep | 0.83 (0.82, 0.85) | 0.89 (0.87, 0.90) | 0.75 (0.72, 0.79) | 0.85 (0.82, 0.89) | 0.86 (0.83, 0.88) | 0.89 (0.86, 0.91) |
| Frequent social connection | 0.94 (0.92, 0.96) | 0.96 (0.94, 0.98) | 0.81 (0.77, 0.85) | 0.87 (0.82, 0.92) | 0.75 (0.72, 0.78) | 0.78 (0.76, 0.81) |

Note: FCRMD: first cardio-renal-metabolic disease; CRMM: cardio-renal-metabolic multimorbidity (the coexistence of two or three CRMDs after FCRMD). CVD: cardiovascular disease, T2D: type 2 diabetes, CKD: chronic kidney disease. Model 1 was adjusted for age and sex. Model 2 was further adjusted for race, Townsend deprivation index, education, BMI, and assessment centers. For analyses on individual lifestyle factors, all lifestyle factors were simultaneously adjusted. All P values were < 0.001.

**Table S5. Associations of lifestyle with the risks of FCRMD, CRMM, and death using multi-state model.**


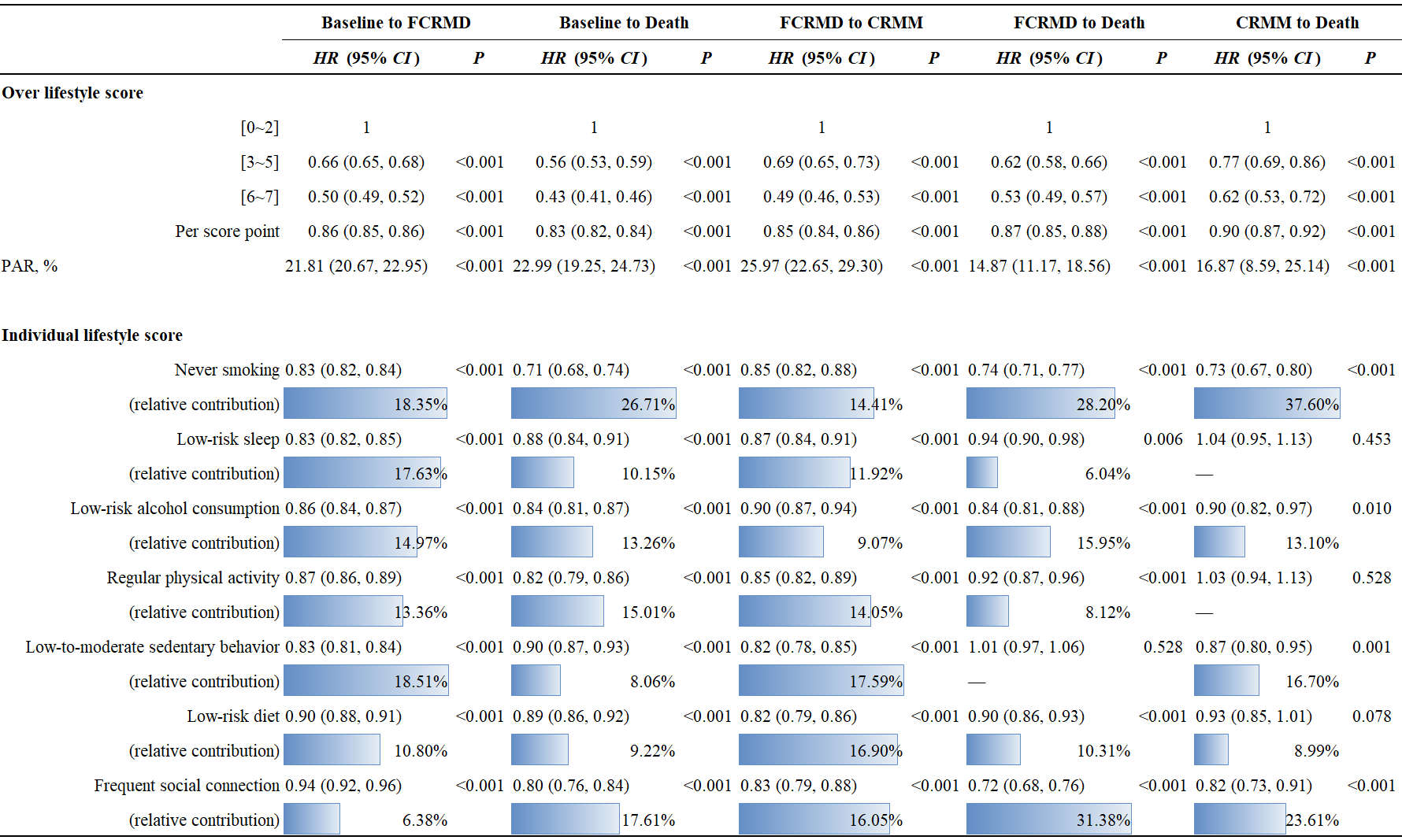


Note: HR: hazard ratio, CI: confidence interval, FCRMD: first cardio-renal-metabolic disease; CRMM: cardio-renal-metabolic multimorbidity (the coexistence of two or three CRMDs after FCRMD), CVD: cardiovascular disease, T2D: type 2 diabetes, CKD: chronic kidney disease. Model was adjusted for age and sex. Relative contribution estimations of lifestyle factors with the disease dynamic progression were using quantile G-computation. Blue bars represented relative contributions or weights of lifestyle factors. Model 1 was adjusted for age and sex.

**Table S6. The results of stratified analyses.**

| **Transition** | ***HR* (95% *CI*)** | | ***P* for interaction** |
| --- | --- | --- | --- |
| **Age** | **Young** | **Old** |  |
| Baseline to FCRMD | 0.89 (0.88, 0.90) | 0.92 (0.91, 0.92) | <0.001 |
| Baseline to Death | 0.82 (0.80, 0.84) | 0.85 (0.84, 0.87) | <0.001 |
| FCRMD to CRMM | 0.88 (0.85, 0.90) | 0.91 (0.89, 0.92) | 0.013 |
| FCRMD to Death | 0.87 (0.83, 0.90) | 0.87 (0.86, 0.89) | 0.428 |
| CRMM to Death | 0.93 (0.85, 1.01) | 0.90 (0.87, 0.93) | 0.998 |
| **Sex** | **Male** | **Female** |  |
| Baseline to FCRMD | 0.92 (0.91, 0.92) | 0.90 (0.89, 0.91) | 0.002 |
| Baseline to Death | 0.83 (0.82, 0.85) | 0.86 (0.84, 0.87) | 0.004 |
| FCRMD to CRMM | 0.91 (0.89, 0.93) | 0.89 (0.87, 0.91) | 0.858 |
| FCRMD to Death | 0.86 (0.85, 0.88) | 0.89 (0.86, 0.91) | 0.022 |
| CRMM to Death | 0.93 (0.89, 0.96) | 0.87 (0.83, 0.91) | 0.015 |
| **SES** | **Low SES** | **High SES** |  |
| Baseline to FCRMD | 0.89 (0.89, 0.90) | 0.93 (0.92, 0.94) | <0.001 |
| Baseline to Death | 0.83 (0.81, 0.84) | 0.87 (0.85, 0.88) | <0.001 |
| FCRMD to CRMM | 0.90 (0.88, 0.92) | 0.91 (0.89, 0.93) | 0.888 |
| FCRMD to Death | 0.85 (0.84, 0.87) | 0.90 (0.88, 0.92) | 0.001 |
| CRMM to Death | 0.91 (0.88, 0.95) | 0.89 (0.85, 0.93) | 0.595 |

Note: HR: hazard ratio, CI: confidence interval, FCRMD: first cardio-renal-metabolic disease; CRMM: cardio-renal-metabolic multimorbidity (the coexistence of two or three CRMDs after FCRMD), SES: Socioeconomic status. We used Townsend deprivation index (TDI) as the SES variable, with low SES defined as TDI > its median, and high SES defined as TDI ≤ its median^16^.

**Table S7. Association of lifestyle score with the disease progression, when constructing lifestyle score with four conventional factors.**

| **Over lifestyle score** | **Baseline to FCRMD** | | **Baseline to Death** | | **FCRMD to CRMM** | | **FCRMD to Death** | | **CRMM to Death** | |
| --- | --- | --- | --- | --- | --- | --- | --- | --- | --- | --- |
|  | ***HR* (95% *CI*)** | ***P*** | ***HR* (95% *CI*)** | ***P*** | ***HR* (95% *CI*)** | ***P*** | ***HR* (95% *CI*)** | ***P*** | ***HR* (95% *CI*)** | ***P*** |
| [0~2] | 1 |  | 1 |  | 1 |  | 1 |  | 1 |  |
| [3~5] | 0.81 (0.80, 0.83) | <0.001 | 0.68 (0.65, 0.70) | <0.001 | 0.80 (0.77, 0.84) | <0.001 | 0.71 (0.68, 0.74) | <0.001 | 0.80 (0.73, 0.87) | <0.001 |
| [6~7] | 0.73 (0.71, 0.75) | <0.001 | 0.56 (0.52, 0.59) | <0.001 | 0.69 (0.64, 0.74) | <0.001 | 0.60 (0.55, 0.65) | <0.001 | 0.72 (0.61, 0.85) | <0.001 |
| Per score point | 0.90 (0.89, 0.90) | <0.001 | 0.82 (0.80, 0.83) | <0.001 | 0.89 (0.87, 0.90) | <0.001 | 0.84 (0.83, 0.86) | <0.001 | 0.89 (0.85, 0.92) | <0.001 |

Note: HR: hazard ratio, CI: confidence interval, FCRMD: first cardio-renal-metabolic disease; CRMM: cardio-renal-metabolic multimorbidity (the coexistence of two or three CRMDs after FCRMD).

**Table S8. Association of lifestyle score with the disease progression, when including body mass index in the lifestyle score.**

| **Over lifestyle score** | **Baseline to FCRMD** | | **Baseline to Death** | | **FCRMD to CRMM** | | **FCRMD to Death** | | **CRMM to Death** | |
| --- | --- | --- | --- | --- | --- | --- | --- | --- | --- | --- |
|  | ***HR* (95% *CI*)** | ***P*** | ***HR* (95% *CI*)** | ***P*** | ***HR* (95% *CI*)** | ***P*** | ***HR* (95% *CI*)** | ***P*** | ***HR* (95% *CI*)** | ***P*** |
| [0~2] | 1 |  | 1 |  | 1 |  | 1 |  | 1 |  |
| [3~5] | 0.70 (0.68, 0.71) | <0.001 | 0.65 (0.61, 0.69) | <0.001 | 0.71 (0.67, 0.75) | <0.001 | 0.73 (0.68, 0.78) | <0.001 | 0.83 (0.74, 0.93) | 0.002 |
| [6~7] | 0.50 (0.49, 0.52) | <0.001 | 0.51 (0.48, 0.55) | <0.001 | 0.49 (0.46, 0.52) | <0.001 | 0.66 (0.61, 0.71) | <0.001 | 0.71 (0.61, 0.83) | <0.001 |
| Per score point | 0.86 (0.86, 0.87) | <0.001 | 0.88 (0.87, 0.89) | <0.001 | 0.85 (0.84, 0.86) | <0.001 | 0.92 (0.90, 0.93) | <0.001 | 0.93 (0.90, 0.96) | <0.001 |

Note: HR: hazard ratio, CI: confidence interval, FCRMD: first cardio-renal-metabolic disease; CRMM: cardio-renal-metabolic multimorbidity (the coexistence of two or three CRMDs after FCRMD).

**Table S9. Association of lifestyle score with the disease progression, redefining low-risk alcohol consumption as no heavy drinking.**

| **Over lifestyle score** | **Baseline to FCRMD** | | **Baseline to Death** | | **FCRMD to CRMM** | | **FCRMD to Death** | | **CRMM to Death** | |
| --- | --- | --- | --- | --- | --- | --- | --- | --- | --- | --- |
|  | ***HR* (95% *CI*)** | ***P*** | ***HR* (95% *CI*)** | ***P*** | ***HR* (95% *CI*)** | ***P*** | ***HR* (95% *CI*)** | ***P*** | ***HR* (95% *CI*)** | ***P*** |
| [0~2] | 1 |  | 1 |  | 1 |  | 1 |  | 1 |  |
| [3~5] | 0.79 (0.77, 0.82) | <0.001 | 0.59 (0.56, 0.63) | <0.001 | 0.81 (0.76, 0.86) | <0.001 | 0.65 (0.61, 0.70) | <0.001 | 0.76 (0.67, 0.86) | <0.001 |
| [6~7] | 0.70 (0.67, 0.72) | <0.001 | 0.49 (0.46, 0.53) | <0.001 | 0.70 (0.65, 0.76) | <0.001 | 0.56 (0.52, 0.61) | <0.001 | 0.64 (0.55, 0.75) | <0.001 |
| Per score point | 0.92 (0.92, 0.93) | <0.001 | 0.86 (0.84, 0.87) | <0.001 | 0.92 (0.90, 0.93) | <0.001 | 0.88 (0.86, 0.89) | <0.001 | 0.90 (0.88, 0.93) | <0.001 |

Note: HR: hazard ratio, CI: confidence interval, FCRMD: first cardio-renal-metabolic disease; CRMM: cardio-renal-metabolic multimorbidity (the coexistence of two or three CRMDs after FCRMD).

**Table S10. Association of lifestyle score with the disease progression, when additionally adjusting for health-related variables.**

| **Over lifestyle score** | **Baseline to FCRMD** | | **Baseline to Death** | | **FCRMD to CRMM** | | **FCRMD to Death** | | **CRMM to Death** | |
| --- | --- | --- | --- | --- | --- | --- | --- | --- | --- | --- |
|  | ***HR* (95% *CI*)** | ***P*** | ***HR* (95% *CI*)** | ***P*** | ***HR* (95% *CI*)** | ***P*** | ***HR* (95% *CI*)** | ***P*** | ***HR* (95% *CI*)** | ***P*** |
| [0~2] | 1 |  | 1 |  | 1 |  | 1 |  | 1 |  |
| [3~5] | 0.83 (0.81, 0.85) | <0.001 | 0.66 (0.62, 0.70) | <0.001 | 0.84 (0.79, 0.89) | <0.001 | 0.69 (0.65, 0.73) | <0.001 | 0.83 (0.75, 0.93) | 0.002 |
| [6~7] | 0.74 (0.72, 0.76) | <0.001 | 0.56 (0.52, 0.60) | <0.001 | 0.72 (0.67, 0.78) | <0.001 | 0.61 (0.56, 0.66) | <0.001 | 0.69 (0.58, 0.80) | <0.001 |
| Per score point | 0.94 (0.93, 0.94) | <0.001 | 0.88 (0.87, 0.89) | <0.001 | 0.92 (0.91, 0.94) | <0.001 | 0.89 (0.87, 0.90) | <0.001 | 0.92 (0.89, 0.95) | <0.001 |

Note: HR: hazard ratio, CI: confidence interval, FCRMD: first cardio-renal-metabolic disease; CRMM: cardio-renal-metabolic multimorbidity (the coexistence of two or three CRMDs after FCRMD).

**Table S11. Association of lifestyle score with the disease dynamic progression, when additionally adjusting for baseline systolic blood pressure, diastolic blood pressure and cholesterol.**

| **Over lifestyle score** | **Baseline to FCRMD** | | **Baseline to Death** | | **FCRMD to CRMM** | | **FCRMD to Death** | | **CRMM to Death** | |
| --- | --- | --- | --- | --- | --- | --- | --- | --- | --- | --- |
|  | ***HR* (95% *CI*)** | ***P*** | ***HR* (95% *CI*)** | ***P*** | ***HR* (95% *CI*)** | ***P*** | ***HR* (95% *CI*)** | ***P*** | ***HR* (95% *CI*)** | ***P*** |
| [0~2] | 1 |  | 1 |  | 1 |  | 1 |  | 1 |  |
| [3~5] | 0.76 (0.74, 0.78) | <0.001 | 0.59 (0.55, 0.62) | <0.001 | 0.78 (0.74, 0.83) | <0.001 | 0.64 (0.60, 0.68) | 0 | 0.78 (0.70, 0.87) | <0.001 |
| [6~7] | 0.65 (0.63, 0.67) | <0.001 | 0.46 (0.43, 0.50) | <0.001 | 0.64 (0.60, 0.69) | <0.001 | 0.55 (0.51, 0.59) | 0 | 0.62 (0.53, 0.73) | <0.001 |
| Per score point | 0.91 (0.90, 0.91) | <0.001 | 0.84 (0.83, 0.85) | <0.001 | 0.90 (0.89, 0.91) | <0.001 | 0.87 (0.86, 0.88) | 0 | 0.90 (0.87, 0.93) | <0.001 |

Note: HR: hazard ratio, CI: confidence interval, FCRMD: first cardio-renal-metabolic disease; CRMM: cardio-renal-metabolic multimorbidity (the coexistence of two or three CRMDs after FCRMD).

**Table S12.** **Association of lifestyle score with the disease progression, excluding outcome events that occurred in the first six months of follow-up.**

| **Over lifestyle score** | **Baseline to FCRMD** | | **Baseline to Death** | | **FCRMD to CRMM** | | **FCRMD to Death** | | **CRMM to Death** | |
| --- | --- | --- | --- | --- | --- | --- | --- | --- | --- | --- |
|  | ***HR* (95% *CI*)** | ***P*** | ***HR* (95% *CI*)** | ***P*** | ***HR* (95% *CI*)** | ***P*** | ***HR* (95% *CI*)** | ***P*** | ***HR* (95% *CI*)** | ***P*** |
| [0~2] | 1 |  | 1 |  | 1 |  | 1 |  | 1 |  |
| [3~5] | 0.76 (0.74, 0.78) | <0.001 | 0.59 (0.56, 0.62) | <0.001 | 0.77 (0.73, 0.82) | <0.001 | 0.65 (0.61, 0.69) | <0.001 | 0.80 (0.72, 0.90) | <0.001 |
| [6~7] | 0.65 (0.63, 0.67) | <0.001 | 0.47 (0.44, 0.50) | <0.001 | 0.63 (0.59, 0.68) | <0.001 | 0.55 (0.51, 0.60) | <0.001 | 0.65 (0.55, 0.76) | <0.001 |
| Per score point | 0.91 (0.90, 0.91) | <0.001 | 0.85 (0.84, 0.86) | <0.001 | 0.90 (0.88, 0.91) | <0.001 | 0.87 (0.86, 0.89) | <0.001 | 0.91 (0.88, 0.94) | <0.001 |

Note: HR: hazard ratio, CI: confidence interval, FCRMD: first cardio-renal-metabolic disease; CRMM: cardio-renal-metabolic multimorbidity (the coexistence of two or three CRMDs after FCRMD).

**Table S13. Association of lifestyle score with the disease progression, using different intervals for the participants entering different states on the same date.**

| **Disease dynamic progress** | **1 day** | | **30 days** | | **365 days** | |
| --- | --- | --- | --- | --- | --- | --- |
|  | ***HR* (95% *CI*)** | ***P*** | ***HR* (95% *CI*)** | ***P*** | ***HR* (95% *CI*)** | ***P*** |
| Baseline to FCRMD | 0.9092 (0.9041, 0.9143) | <0.001 | 0.9092 (0.9040, 0.9143) | <0.001 | 0.9092 (0.9040, 0.9143) | <0.001 |
| Baseline to Death | 0.8448 (0.8337, 0.8560) | <0.001 | 0.8448 (0.8337, 0.8560) | <0.001 | 0.8446 (0.8335, 0.8558) | <0.001 |
| FCRMD to CRMM | 0.9001 (0.8872, 0.9132) | <0.001 | 0.9001 (0.8872, 0.9132) | <0.001 | 0.9013 (0.8884, 0.9144) | <0.001 |
| FCRMD to Death | 0.8719 (0.8585, 0.8854) | <0.001 | 0.8720 (0.8587, 0.8855) | <0.001 | 0.8723 (0.8589, 0.8859) | <0.001 |
| CRMM to Death | 0.9038 (0.8768, 0.9317) | <0.001 | 0.9036 (0.8766, 0.9315) | <0.001 | 0.9042 (0.8771, 0.9322) | <0.001 |

Note: HR: hazard ratio, CI: confidence interval, FCRMD: first cardio-renal-metabolic disease; CRMM: cardio-renal-metabolic multimorbidity (the coexistence of two or three CRMDs after FCRMD).

**Table S14. Association of lifestyle score with progression from baseline to FCRMD, two CRMM, three CRMM, and ultimately to death.**

| **Disease dynamic progress** | ***HR* (95% *CI*)** | ***P*** |
| --- | --- | --- |
| **Baseline to FCRMD** |  |  |
| Unfavorable [0-2] | 1 |  |
| Intermediate [3-5] | 0.76 (0.74, 0.78) | <0.001 |
| Positive [6-7] | 0.65 (0.63, 0.67) | <0.001 |
| **Baseline to Death** |  |  |
| Unfavorable [0-2] | 1 |  |
| Intermediate [3-5] | 0.59 (0.55, 0.62) | <0.001 |
| Positive [6-7] | 0.47 (0.44, 0.50) | <0.001 |
| **FCRMD to Two CRMM** |  |  |
| Unfavorable [0-2] | 1 |  |
| Intermediate [3-5] | 0.78 (0.74, 0.82) | <0.001 |
| Positive [6-7] | 0.64 (0.60, 0.69) | <0.001 |
| **FCRMD to Death** |  |  |
| Unfavorable [0-2] | 1 |  |
| Intermediate [3-5] | 0.65 (0.61, 0.69) | <0.001 |
| Positive [6-7] | 0.55 (0.51, 0.60) | <0.001 |
| **Two CRMM to Three CRMM** |  |  |
| Unfavorable [0-2] | 1 |  |
| Intermediate [3-5] | 0.80 (0.67, 0.95) | 0.012 |
| Positive [6-7] | 0.66 (0.52, 0.85) | 0.001 |
| **Two CRMM to Death** |  |  |
| Unfavorable [0-2] | 1 |  |
| Intermediate [3-5] | 0.81 (0.72, 0.92) | 0.001 |
| Positive [6-7] | 0.67 (0.57, 0.79) | <0.001 |
| **Three CRMM to Death** |  |  |
| Unfavorable [0-2] | 1 |  |
| Intermediate [3-5] | 0.72 (0.53, 0.99) | 0.045 |
| Positive [6-7] | 0.48 (0.28, 0.82) | 0.007 |
|  |  |  |
| **Per score point** |  |  |
| Baseline to FCRMD | 0.91 (0.90, 0.91) | <0.001 |
| Baseline to Death | 0.84 (0.83, 0.86) | <0.001 |
| FCRMD to Two CRMM | 0.90 (0.89, 0.91) | <0.001 |
| FCRMD to Death | 0.87 (0.86, 0.89) | <0.001 |
| Two CRMM to Three CRMM | 0.91 (0.87, 0.96) | <0.001 |
| Two CRMM to Death | 0.91 (0.88, 0.94) | <0.001 |
| Three CRMM to Death | 0.87 (0.79, 0.96) | 0.005 |

Note: HR: hazard ratio, CI: confidence interval, FCRMD: first cardio-renal-metabolic disease; CRMM: cardio-renal-metabolic multimorbidity (the coexistence of two or three CRMDs after FCRMD).

**Table S15. Association of lifestyle score with progression from baseline to one of CVD, T2D, CKD, then to cardio-renal-metabolic multimorbidity (CRMM), and ultimately to death.**

| **Disease dynamic progress** | ***HR* (95% *CI*)** | ***P* value** |
| --- | --- | --- |
| **Baseline to CVD** |  |  |
| Unfavorable [0-2] | 1 |  |
| Intermediate [3-5] | 0.78 (0.76, 0.80) | <0.001 |
| Positive [6-7] | 0.69 (0.66, 0.71) | <0.001 |
| **Baseline to T2D** |  |  |
| Unfavorable [0-2] | 1 |  |
| Intermediate [3-5] | 0.71 (0.67, 0.75) | <0.001 |
| Positive [6-7] | 0.51 (0.48, 0.55) | <0.001 |
| **Baseline to CKD** |  |  |
| Unfavorable [0-2] | 1 | NA |
| Intermediate [3-5] | 0.73 (0.68, 0.79) | <0.001 |
| Positive [6-7] | 0.63 (0.58, 0.69) | <0.001 |
| **Baseline to Death** |  |  |
| Unfavorable [0-2] | 1 |  |
| Intermediate [3-5] | 0.58 (0.55, 0.62) | <0.001 |
| Positive [6-7] | 0.47 (0.44, 0.50) | <0.001 |
| **CVD to CRMM** |  |  |
| Unfavorable [0-2] | 1 |  |
| Intermediate [3-5] | 0.78 (0.71, 0.85) | <0.001 |
| Positive [6-7] | 0.62 (0.56, 0.70) | <0.001 |
| **CVD to Death** |  |  |
| Unfavorable [0-2] | 1 |  |
| Intermediate [3-5] | 0.62 (0.58, 0.67) | <0.001 |
| Positive [6-7] | 0.53 (0.48, 0.57) | <0.001 |
| **T2D to CRMM** |  |  |
| Unfavorable [0-2] | 1 |  |
| Intermediate [3-5] | 0.85 (0.76, 0.96) | 0.01 |
| Positive [6-7] | 0.78 (0.65, 0.92) | 0.004 |
| **T2D to Death** |  |  |
| Unfavorable [0-2] | 1 |  |
| Intermediate [3-5] | 0.75 (0.62, 0.90) | 0.003 |
| Positive [6-7] | 0.71 (0.55, 0.92) | 0.009 |
| **CKD to CRMM** |  |  |
| Unfavorable [0-2] | 1 |  |
| Intermediate [3-5] | 0.69 (0.60, 0.80) | <0.001 |
| Positive [6-7] | 0.58 (0.48, 0.69) | <0.001 |
| **CKD to Death** |  |  |
| Unfavorable [0-2] | 1 |  |
| Intermediate [3-5] | 0.64 (0.52, 0.80) | <0.001 |
| Positive [6-7] | 0.58 (0.44, 0.76) | <0.001 |
| **CRMM to Death** |  |  |
| Unfavorable [0-2] | 1 |  |
| Intermediate [3-5] | 0.76 (0.67, 0.86) | <0.001 |
| Positive [6-7] | 0.58 (0.48, 0.69) | <0.001 |
|  |  |  |
| **Per score point** |  |  |
| Baseline to CVD | 0.92 (0.91, 0.93) | <0.001 |
| Baseline to T2D | 0.86 (0.85, 0.87) | <0.001 |
| Baseline to CKD | 0.91 (0.89, 0.92) | <0.001 |
| Baseline to Death | 0.84 (0.83, 0.86) | <0.001 |
| CVD to CRMM | 0.90 (0.88, 0.92) | <0.001 |
| CVD to Death | 0.86 (0.85, 0.87) | <0.001 |
| T2D to CRMM | 0.94 (0.91, 0.97) | <0.001 |
| T2D to Death | 0.91 (0.87, 0.96) | 0.001 |
| CKD to CRMM | 0.89 (0.86, 0.92) | <0.001 |
| CKD to Death | 0.91 (0.86, 0.96) | 0.001 |
| CRMM to Death | 0.89 (0.86, 0.92) | <0.001 |

Note: HR: hazard ratio, CI: confidence interval, CVD: cardiovascular disease, T2D: type 2 diabetes, CKD: chronic kidney disease, CRMM: cardio-renal-metabolic multimorbidity (the coexistence of two or three CRMDs after FCRMD).

References

1. Zhao Y, Yang L, Sahakian BJ, et al. The brain structure, immunometabolic and genetic mechanisms underlying the association between lifestyle and depression. *Nature Mental Health*. 2023/10/01 2023;1(10):736-750. doi:10.1038/s44220-023-00120-1

2. Han H, Cao Y, Feng C, et al. Association of a Healthy Lifestyle With All-Cause and Cause-Specific Mortality Among Individuals With Type 2 Diabetes: A Prospective Study in UK Biobank. *Diabetes Care*. Feb 1 2022;45(2):319-329. doi:10.2337/dc21-1512

3. Wang B, Wang N, Sun Y, Tan X, Zhang J, Lu Y. Association of Combined Healthy Lifestyle Factors With Incident Dementia in Patients With Type 2 Diabetes. *Neurology*. Nov 22 2022;99(21):e2336-e2345. doi:10.1212/wnl.0000000000201231

4. Phillips JA. *Dietary Guidelines for Americans, 2020–2025*. vol 69. Workplace Health & Safety. 2021:395-395.

5. WHO guidelines on physical activity and sedentary behaviour. WHO, World Health Organization; 2020.

6. Watson NF, Badr MS, Belenky G, et al. Recommended Amount of Sleep for a Healthy Adult: A Joint Consensus Statement of the American Academy of Sleep Medicine and Sleep Research Society. *Sleep*. Jun 1 2015;38(6):843-4. doi:10.5665/sleep.4716

7. Smoking: reducing and preventing tobacco use. NICE, National Institute for Health and Care Excellence. <https://www.nice.org.uk/guidance/qs82>

8. Smith RW, Barnes I, Green J, Reeves GK, Beral V, Floud S. Social isolation and risk of heart disease and stroke: analysis of two large UK prospective studies. *Lancet Public Health*. Apr 2021;6(4):e232-e239. doi:10.1016/S2468-2667(20)30291-7

9. Keil AP, Buckley JP, O'Brien KM, Ferguson KK, Zhao S, White AJ. A Quantile-Based g-Computation Approach to Addressing the Effects of Exposure Mixtures. *Environ Health Perspect*. Apr 2020;128(4):47004. doi:10.1289/ehp5838

10. Li S, Guo B, Jiang Y, et al. Long-term Exposure to Ambient PM2.5 and Its Components Associated With Diabetes: Evidence From a Large Population-Based Cohort From China. *Diabetes Care*. Jan 1 2023;46(1):111-119. doi:10.2337/dc22-1585

11. Yan H, Tang W, Wang L, et al. Ambient PM2.5 Components Are Associated With Bone Strength: Evidence From a China Multi-Ethnic Study. *J Clin Endocrinol Metab*. Dec 21 2023;109(1):197-207. doi:10.1210/clinem/dgad425

12. Keil A. The qgcomp package: g-computation on exposure quantiles. Accessed January 1, 2024. <https://cran.r-project.org/web/packages/qgcomp/vignettes/qgcomp-vignette.html>

13. Ndumele CE, Rangaswami J, Chow SL, et al. Cardiovascular-Kidney-Metabolic Health: A Presidential Advisory From the American Heart Association. *Circulation*. Nov 14 2023;148(20):1606-1635. doi:10.1161/cir.0000000000001184

14. Honigberg MC, Zekavat SM, Pirruccello JP, Natarajan P, Vaduganathan M. Cardiovascular and Kidney Outcomes Across the Glycemic Spectrum: Insights From the UK Biobank. *J Am Coll Cardiol*. Aug 3 2021;78(5):453-464. doi:10.1016/j.jacc.2021.05.004

15. Han Y, Hu Y, Yu C, et al. Lifestyle, cardiometabolic disease, and multimorbidity in a prospective Chinese study. *Eur Heart J*. Sep 7 2021;42(34):3374-3384. doi:10.1093/eurheartj/ehab413

16. Zhang YB, Chen C, Pan XF, et al. Associations of healthy lifestyle and socioeconomic status with mortality and incident cardiovascular disease: two prospective cohort studies. *Bmj*. Apr 14 2021;373:n604. doi:10.1136/bmj.n604
